# Dielectric‐Confinement‐Induced in‐Plane Photoelectric Anisotropy in Isotropic Quasi‐1D γ‐GaS Nanoribbon

**DOI:** 10.1002/advs.75736

**Published:** 2026-05-22

**Authors:** Jiawei Jing, Jiamei Chen, Xing Xin, Peisong Peng, Zhihao Wu, Weiheng Zhong, Hanyu Zhang, Wanfu Shen, Jihua Zhang, Yuwei Shan, Yuanzheng Li, Xingang Zhao, Wei Xin, Fang Wang, Weida Hu, Haiyang Xu, Yichun Liu

**Affiliations:** ^1^ State Key Laboratory of Integrated Optoelectronics, Key Laboratory of UV‐Emitting Materials and Technology of Ministry of Education Northeast Normal University Changchun China; ^2^ State Key Laboratory of Infrared Physics, Shanghai Institute of Technical Physics Chinese Academy of Sciences Shanghai China; ^3^ State Key Laboratory of Precision Measuring Technology and Instruments, School of Precision Instrument and Optoelectronics Engineering Tianjin University Tianjin China; ^4^ Songshan Lake Materials Laboratory Dongguan Guangdong China; ^5^ State Key Laboratory of Luminescence Science and Technology, Chinese Academy of Sciences, Changchun Institute of Optics Fine Mechanics and Physics Changchun China

**Keywords:** equivalent in‐plane anisotropy, geometry‐governed dielectric confinement, isotropic materials, polarized photodetection, quasi‐1D γ‐GaS nanoribbon

## Abstract

The growing demand for polarized photodetection has driven the need for compact, integrated, and multifunctional device architectures. Although one‐dimensional (1D) materials are of significant interest for their innate polarization sensitivity among viable integration strategies, current research has largely overlooked the anisotropy induced by geometric dielectric confinement, remaining mainly focused on optoelectronic anisotropy from lattice symmetry breaking. To bridge the gap, here we investigate geometry‐governed optoelectronic anisotropy in quasi‐1D GaS nanoribbons with intrinsically isotropic atomic structures. Dielectric mismatch between the ribbon and its surroundings leads to a general polarization‐dependent photoresponse during near‐field scattering. Steady‐state and transient spectroscopy further reveal that the dielectric confinement substantially modulates light absorption, phonon scattering, and carrier diffusion. Remarkably, this geometry‐governed anisotropy exhibits sufficient strength and robustness to effectively support applications in areas such as polarized imaging, stress mapping, and visual cryptography. Our work provides fundamental insights into the optoelectronic anisotropy of 1D nanomaterials and offers a rational basis for exciting material properties and optimizing device designs in future polarized photonics.

## Graphical abstract

We investigate the geometry‐governed optoelectronic anisotropy arising from dielectric confinement in quasi‐1D γ‐GaS nanoribbons with intrinsically isotropic atomic structures. Dielectric mismatch between the nanoribbon and its surroundings leads to a general polarization‐dependent photoresponse during near‐field scattering. The dielectric confinement significantly modulates the anisotropy of microscopic photocarrier dynamics. This geometry‐governed anisotropy effectively supports applications related to polarization detection.

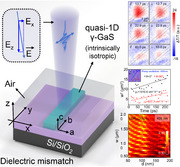

## 1 Introduction

The examination of light polarization plays a pivotal role in advancing modern optoelectronic technologies, offering strategic advantages in enhancing performance across diverse fields, including polarization‐sensitive imaging, optical communication, and artificial vision systems [1, 2, 3]. The growing sophistication of these applications demands unprecedented detection performance of polarization, thereby accelerating the development of related devices toward compact, integrated, and multifunctional architectures [4, 5]. Among emerging solutions, micro/nanomaterial‐based polarized photodetectors have emerged as a distinctive platform, distinguished by superior photoelectric conversion efficiency, heterogeneous integration flexibility, and field‐tunable response characteristics [6, 7]. Specifically, one‐dimensional (1D) materials, characterized by inherent confinement along a single in‐plane axis, demonstrate unique potential in micro/nano‐polarized optoelectronics, positioning them as ideal candidates for high‐performance device structures [8, 9]. The materials usually achieve polarized photoresponse through a dual approach combining intrinsic material anisotropy and performance manipulation via light‐field coupling [9, 10]. For materials with geometric constraints of varying scales, effects like quantum confinement of carriers, dielectrically enhanced exciton behavior, and dielectric contrast all make significant contributions [9]. However, current research predominantly focuses on materials exhibiting intrinsic in‐plane lattice anisotropy (e.g., Sb_2_S_3_, TiS_3_, ZrS_3_), while severely neglecting the anisotropic photoresponse in materials induced by geometric constraints [10, 11, 12, 13, 14]. Despite progress in comprehending geometry‐governed polarization photoelectric modulation in large‐area grating arrays, systematic methodologies for investigations at the single‐nanoribbon level and their technological applications are notably scarce [15, 16]. Addressing this research gap is essential for expanding the material toolkit available for polarized photodetection, overcoming the inherent limitations of traditional anisotropic systems, and unlocking novel functionalities in advanced optoelectronic applications.

Here, we investigate quasi‐1D γ‐GaS nanoribbon as a lattice‐isotropic model system to elucidate the physical mechanisms and technological potential of geometry‐governed polarization‐sensitive photoresponses. Experimentally, we first synthesized γ‐GaS nanoribbon on SiO_2_/Si substrates via physical vapor deposition (PVD), achieving tunable control over dimensional parameters [17, 18]. First‐principle calculations revealed that γ‐GaS, belonging to the *C*_3V_ point group, exhibits negligible quantum confinement effects under this characteristic geometry structure and inherently lacks in‐plane photoelectric anisotropy under illumination [19]. Nevertheless, near‐field electric field simulations demonstrated that the sub‐micrometer‐scale quasi‐1D geometry induces significant equivalent anisotropy through dielectric‐confinement‐induced light scattering [20]. Specifically, normally incident light excites an interfacial in‐plane/out‐of‐plane optical field, creating pronounced disparities in field distribution and absorption cross section (ACS) depending on whether the ribbon's long axis is aligned with or perpendicular to the polarization direction of light. By optimizing the width‐to‐height aspect ratio of the nanoribbon end‐face, the maximal linearly dichroic ratio of light absorption (calculated as differential ACS, *LDR_Abs_*) about 4.50 was theoretically predicted in the visible spectrum. Further experimental validation employed a γ‐GaS nanoribbon with a typical geometry of 0.75 µm width and 110 nm thickness, combined with polarization‐dependent differential reflection spectroscopy, Raman spectroscopy, and transient absorption (TA) spectroscopy, respectively [21, 22, 23]. Mechanistic insights were derived from analyses of phonon vibrational modes and photogenerated carrier diffusion dynamics, establishing a clear link between geometry‐governed dielectric confinement and polarization‐sensitive behaviors. This equivalent anisotropy directly translates to universal polarized photoresponse characteristics in the corresponding γ‐GaS optoelectronic device across a wide spectrum range (320‐532 nm). The measurements revealed a linear dichroism ratio of photoresponse (*LDR_PD_*) of 1.2‐2.1 in this band, and the performance is on par with that of conventional lattice‐anisotropy‐based devices [6, 9]. The implementations in polarized imaging, stress mapping, and visual cryptography were further explored building on these findings [24, 25, 26]. Our work underscores the pivotal role of geometry design in enabling the functional implementation of polarization‐responsive devices while exciting the intrinsic material properties and providing strategic configuration guidelines to advance high‐performance optoelectronic systems.

## 2 Results

### 2.1 Fabrication and Characteristics of Quasi‐1D γ‐GaS Nanoribbons

Layered GaS, a typical III‐VI group chalcogenide material, has garnered significant attention recently due to its promising applications in nonlinear optics, photodetection, and topological photonics [17, 18]. Structurally, monolayer GaS exhibits a characteristic S‐Ga‐Ga‐S atomic sequence along the *c*‐*axis* direction, with individual layers bonded through van der Waals interactions. Four stable polytypic phases, namely β‐, δ‐, ε‐, and γ‐phases, distinguished by their interlayer stacking configurations, have been demonstrated. Among these, the β‐GaS represents the thermodynamically most stable configuration under ambient conditions, while the others can also be mixed‐synthesized due to the small formation energy differences between polymorphs. However, all layered GaS structures exhibit high in‐plane symmetry corresponding to their respective point groups (*D*_6h_, *C*_6v_, *D*_3h_ and *C*_3v_ groups for β‐, δ‐, ε‐, and γ‐GaS, respectively) [19, 27]. This inherent isotropic nature provides a unique opportunity to investigate geometry‐governed anisotropy in optical responses of 1D materials.

**FIGURE 1:**
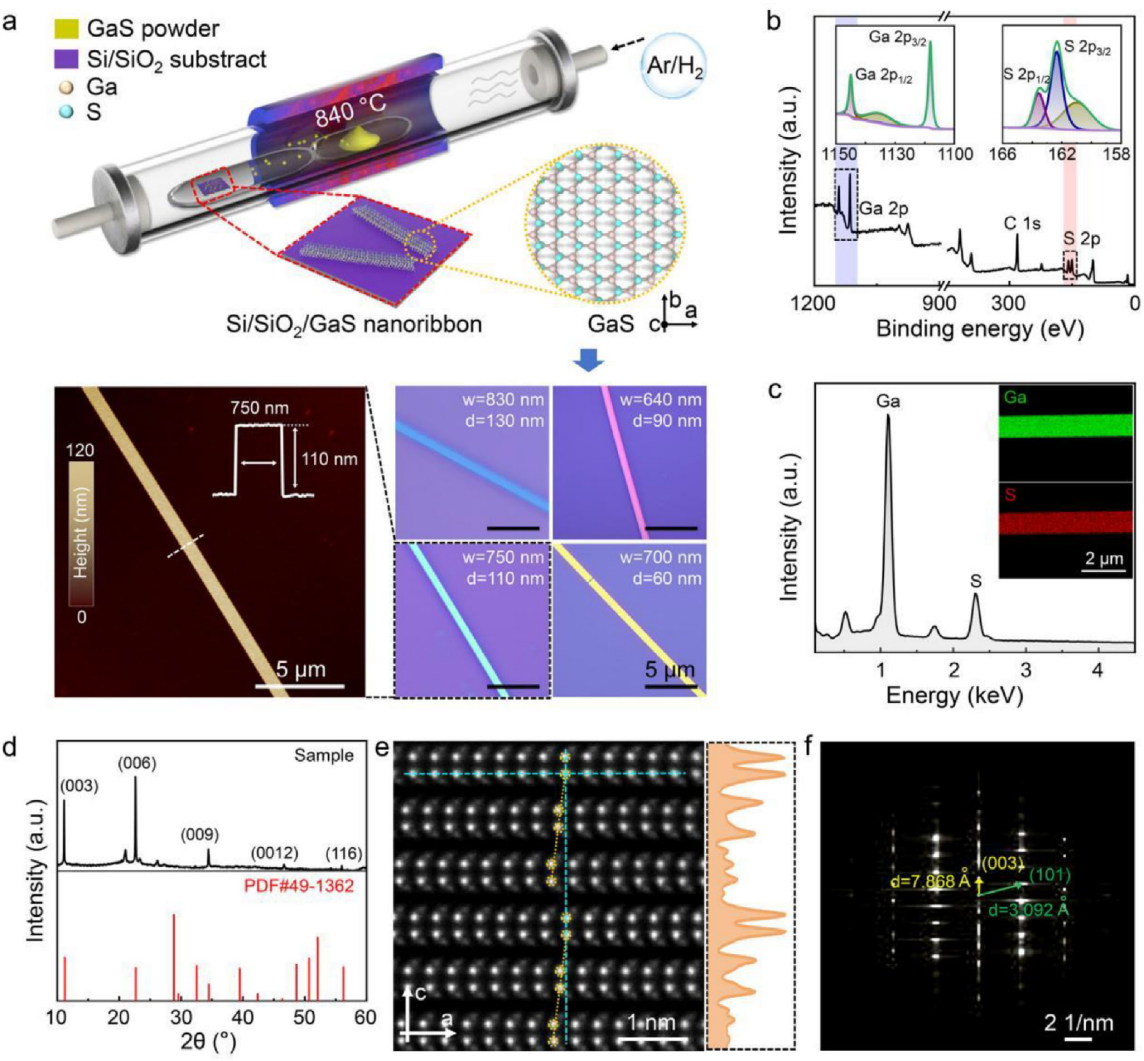
PVD growth and characterization of quasi‐1D γ‐GaS nanoribbons. (a) Schematic diagram, AFM, and optical images of PVD‐grown γ‐GaS materials. The different colors of nanoribbons indicate their different thicknesses. (b–d) XPS, EDS, and XRD of GaS materials. (e, f) HRTEM and FFT image of γ‐GaS materials.

Here, the conventional physical vapor deposition (PVD) technique was employed to synthesize GaS nanoribbons, using commercially sourced GaS powder as the precursor and a hydrogen/argon (H_2_/Ar) mixture as the carrier gas [28]. Following a 7‐min duration at 850°C, the quasi‐1D single‐crystal GaS nanoribbons were successfully grown on SiO_2_/Si substrates (see Methods). Figure 1a presents the schematic synthesis and microscopic morphology images of representative samples. Through precise modulation of growth parameters, including temperature and growth time, the nanoribbon width was systematically tuned from 500 nm to 1.5 µm, while thickness control between 80 nm and 160 nm was achieved, as shown in Sections S1 and S2. Atomic force microscopy (AFM) characterization revealed the typical surface morphology of individual GaS nanoribbons, demonstrating uniform thickness (∼110 nm) and lateral dimensions (∼750 nm width) with exceptionally smooth surfaces free of detectable contaminants or structural defects (Figure 1a).

Following material synthesis, X‐ray photoelectron spectroscopy (XPS, Figure 1b) was promptly employed to analyze the chemical composition of the as‐grown GaS nanoribbons [29]. Charge correction was performed using the C 1s peak (284.8 eV) as a reference, revealing distinct Ga 2p and S 2p spectra within the 0–1200 eV survey range (Figure 1b). Specifically, the Ga 2p_3_/_2_ and Ga 2p₁/_2_ peaks were observed at ∼1118.1 and ∼1145.0 eV, respectively, yielding a spin‐orbit splitting energy of 26.9 eV that aligns precisely with the characteristic value for Ga(III) compounds. The binding energy exhibits a positive shift of ∼1.4 eV compared to metallic Ga (Ga 2p_3_/_2_ peak at ∼1116.7 eV), confirming the +2 oxidation state of Ga through electron transfer to neighboring atoms. Concurrently, the S 2p_3/2_ and S 2p_1/2_ peaks at ∼162.3 and ∼163.6 eV, respectively, showed a spin‐orbit splitting of 1.3 eV, consistent with the spectral signature of sulfide species. These values are significantly lower than elemental sulfur (S 2p_3/2_ peak at ∼164.0 eV) and sulfates (>168 eV), while falling within the 160–163.5 eV range characteristic of S^2−^ species, thus verifying the divalent negative oxidation state of sulfur. This compositional profile matches precisely with the stoichiometric GaS reference, effectively excluding alternative phases such as Ga_2_S_3_ (Ga 2p_3_/_2_ peak less than 1118 eV) or Ga_2_O_3_ (Ga 2p_3_/_2_ peak around 1119.5–1120.5 eV). Complementary energy‐dispersive X‐ray spectroscopy (EDS) elemental mapping (Figure 1c) further corroborated these findings, revealing uniform Ga and S distribution across the nanoribbons with a measured mass ratio of 69.72:32.07 (Ga:S), corresponding to a around 1:1 stoichiometric ratio. The homogeneous elemental distribution observed in mapping images (Figure 1c) attests to the exceptional material quality and compositional uniformity achieved through our synthesis protocol.

To further elucidate the crystalline structure of the synthesized GaS nanoribbons, X‐ray diffraction (XRD) analysis was performed using a 2θ scanning range of 10° to 60° with a step size of 0.02° per second [17]. Figure 1d (upper panel) presents the XRD pattern, revealing distinct diffraction peaks at 11.24°, 22.70°, 34.52°, and 46.36°, corresponding to the (003), (006), (009), and (0012) crystallographic planes of the trigonal system of GaS. These peak positions demonstrate excellent agreement with the standard PDF card (#49‐1362, Figure 1d lower panel), confirming the γ‐GaS structure with lattice parameters a = b = 3.601 Å, c = 23.380 Å, and R3m space group symmetry (details of the peak analysis can be found in Section S3). In addition, the high‐resolution transmission electron microscopy (HRTEM) analysis of the cross‐sectional structure (Figure 1e) also revealed a periodic interlayer sliding pattern where every three GaS layers form a unit cycle along the *c* *‐axis*, consistent with the characteristic stacking sequence of γ‐GaS. The corresponding fast Fourier transform (FFT, Figure 1f) pattern exhibited well‐defined diffraction spots with a measured lattice spacing of 7.868 Å, matching the (003) plane spacing of γ‐GaS. The planar‐view TEM imaging revealed that the nanoribbons exhibit zigzag (ZZ) edges aligned precisely along the defined *b‐axis* direction, with sharp material‐air interfaces confirming the high crystalline quality and single‐domain nature of the samples [17]. Detailed crystallographic analyses are also provided in Section S3, and please refer to the Method section for more information on material characterizations.

### 2.2 Theoretical Insights into Equivalent in‐Plane Optical Anisotropy

**FIGURE 2:**
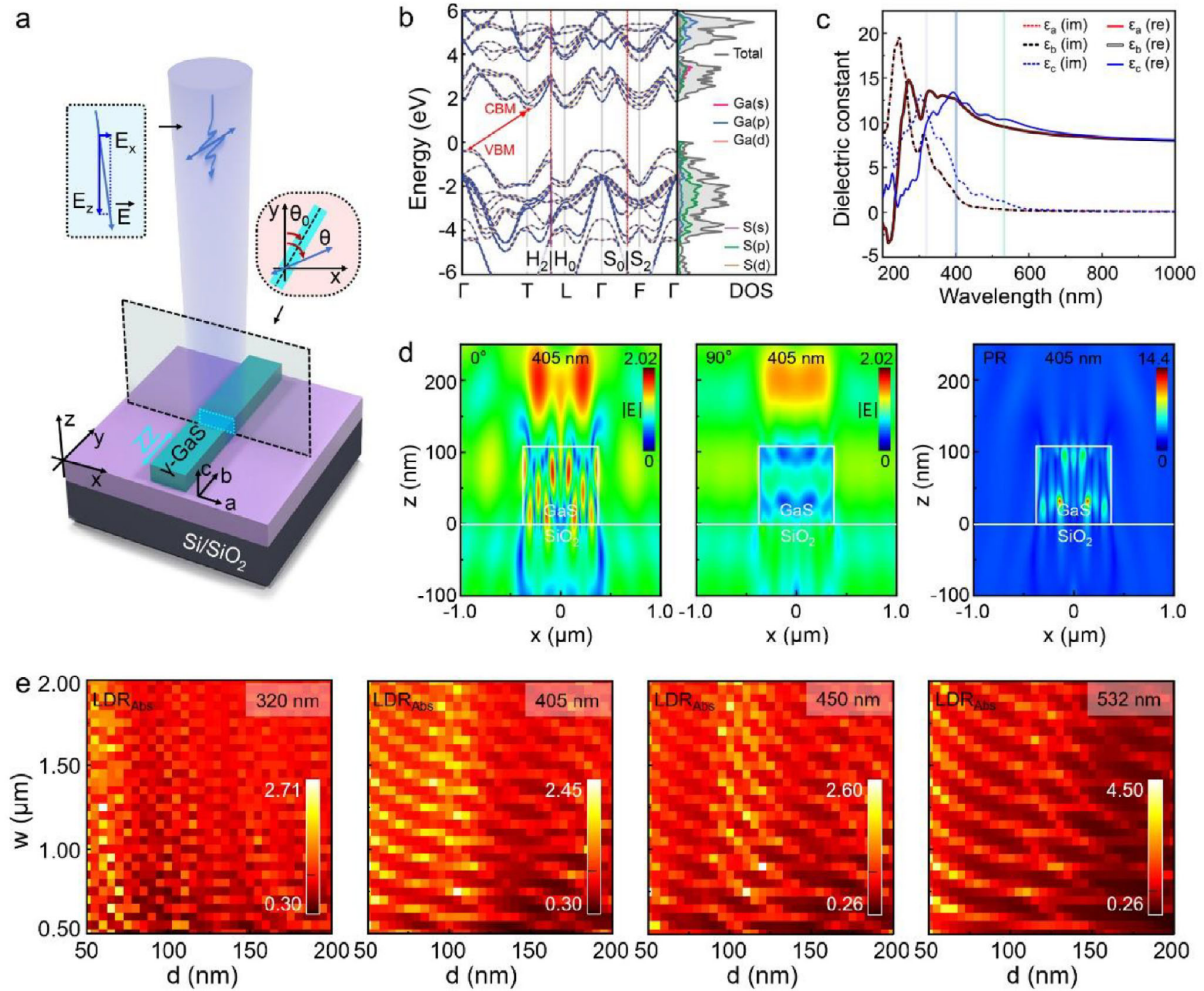
Theoretical exploration of equivalent in‐plane optical anisotropy of γ‐GaS nanoribbons. (a) Schematic diagram of a GaS nanoribbon under polarized light excitation. (b) Band structure of bulk γ‐GaS. (c) Calculated real and imaginary components of the dielectric constant along the *a*‐, *b*‐, and *c‐axes* of GaS nanoribbon. (d) Anisotropic near‐field electric field intensity distributions and ACS in the x‐z plane of GaS nanoribbons under 405 nm light illumination with s‐ and p‐polarizations. (e) *LDR_Abs_* of ribbons with different geometry parameters under illumination at wavelengths of 320, 405, 450, and 532 nm.

After confirming the synthesized quasi‐1D γ‐GaS nanoribbons, we conducted a theoretical investigation into the geometry‐governed in‐plane optical anisotropy under normal light incidence. A model was established in Figure 2a, where the crystal axes were mapped to a custom‐defined coordinate system. The *c* *‐axis* of the GaS nanoribbon is posited parallel to the *z* *‐axis*, while the mutually perpendicular *a*‐ and *b‐axes* of the sample lie within the same plane as the *x‐* and *y‐axes*. We further define the *y* *‐axis* as the fundamental reference, and the *θ* (*θ*_0_) represents the angle measured between the polarization state of incident light (*b*‐*axis* of the ribbon) and the *y* *‐axis* after undergoing a clockwise rotation from the initial direction. Unless explicitly stated otherwise, we generally assume that the *b‐axis* of the GaS nanoribbon is aligned parallel to the *y‐axis*, thereby implying that the angle *θ*_0_ is zero.

Then, we calculated the electronic band structure of bulk γ‐GaS along the high‐symmetry path using density functional theory (DFT), as shown in Section S4. The results revealed an indirect bandgap configuration, with the valence band maximum (VBM) and conduction band minimum (CBM) located at distinct k‐points, yielding a calculated indirect bandgap of 1.79 eV (Figure 2b). Notably, this value underestimates the experimentally measured bandgap of 2.53 eV (Section S5), attributable to the use of semilocal exchange‐correlation functionals in the DFT framework [30]. Subsequent analysis of the electronic density of states (DOS) demonstrated that the VBM is predominantly composed of Ga 3p and S 3p orbital hybridizations, while the CBM is primarily derived from Ga 4s and S 3p orbital contributions. The partial charge density (PCDS) distributions at the VBM and CBM (Figure S4c) exhibit striking correspondence with the orbital contribution analysis, where the spatial characteristics of PCDS validate the orbital hybridization mechanisms governing valence band formation and conduction band character [31].

The calculation also provides predicted properties for γ‐GaS, revealing that within the a‐b plane, the material exhibits intrinsic optical isotropy with strong consistency between the real/imaginary parts of its permittivity (Figure 2c and Figure S4b). In contrast, marked disparities emerge between in‐plane (a, b) and out‐of‐plane (*c*‐*axis*) directions for both dielectric constant and absorption characteristics. That means, under ideal conditions, the incident light irradiated normally on quasi‐1D γ‐GaS nanoribbons would not induce significant in‐plane photoelectric anisotropy due to the intrinsic material properties. However, the nanoribbon's unique 1D geometry introduces discrepant dielectric confinement between the material and its surroundings, leading to different interfacial in‐plane light scattering. The discrepancy in optical coupling efficiency among polarized lights ultimately results in emergent equivalent anisotropy.

To verify the aforementioned conclusion, we conducted another analysis of the electromagnetic response of the model under light excitation by using the finite element (FEM) method [6, 14]. Figure 2d illustrates the near‐field electric field intensity (|*E*|) distributions at the x‐z plane of γ‐GaS nanoribbons (750 nm width, 110 nm thickness) under s‐polarized and p‐polarized illumination of 405 nm wavelength. The |*E*| distribution within the nanoribbon exhibits relative uniformity under p‐polarized illumination, while the s‐polarized illumination induces significant |*E*| enhancement. This disparity, visualized through the ratio of spatial electric field intensities (Figure 2d right), directly contributes to the in‐plane optical anisotropy. For quantitative characterization, we extracted the absorption cross‐section (ACS) in the x‐z plane of the nanoribbon under light excitation with two different polarization states. The ACS, defined as the material's absorption capacity in the x‐z plane per unit length along the *y*‐*axis*, yielded values of 2.10 × 10^−10^ m for s‐polarized and 1.16 × 10^−10^ m for p‐polarized illumination, with an anisotropy ratio of ∼1.81 (differential ACS, marked as *LDR_Abs_*). The numerical difference quantifies the material's differential absorption efficiency of orthogonal‐polarized light.

This geometry‐governed in‐plane anisotropy of GaS demonstrates certain universality. Simulations across multiple wavelengths confirm similar material behaviors in Section S6, while systematic variation of nanoribbon dimensions (width of 0.5–1.5 µm, thickness of 80–160 nm) reveals tunable anisotropic characteristics. Contrary to common oversight, a stronger light‐matter interaction in 1D materials does not invariably manifest along their long axis. Figure 2e illustrates the differential ACS trends under 320, 405, 450, and 532 nm polarized illumination as geometric parameters change. The material consistently exhibits anisotropy with *LDR_Abs_* deviating from unity. In the absence of constraints on geometric parameters, the maximum ratio attains a value of 4.50. This is comparable to or exceeds the intrinsic optical anisotropies arising from crystalline lattice asymmetry [6]. Notably, while simulation results establish a universal structural parameter dependence, further experimental investigations primarily focus on 405 nm laser irradiation due to the typical dimensions of our samples (∼0.75 µm in width, ∼110 nm in thickness) and optimized comprehensive photoresponse performance in experiments. It should also be emphasized that the infinite‐length assumption along the *y*‐*axis* in simulations resulted in ACS dimensions expressed in meters rather than conventional square meters [32]. Detailed computational methodologies and parameter analyses are provided in the Method section and Section S6.

### 2.3 In‐Plane Optical Anisotropy Measurements

The above study investigates the mechanisms underlying the emergent equivalent in‐plane anisotropy in the quasi‐1D γ‐GaS nanoribbon via theoretical simulations, focusing on how the geometry‐governed dielectric confinement influences the near‐field distribution of electromagnetic waves and consequently induces anisotropic optical responses. We further analyze the experimental manifestations of such anisotropy through differential reflectance spectroscopy, phonon vibrations, and photogenerated carrier diffusion dynamics, respectively [21, 22, 23]. From a macroscopic perspective, the anisotropy of materials can be directly discerned through variations in spectral information, such as absorption, reflection, and transmission [10]. To explore the characteristic, we employed azimuth‐dependent reflectance difference microscopy (ADRMD) imaging of GaS nanoribbon, an exceptionally effective technique that facilitates non‐destructive in situ optical anisotropy studies [33]. The test results reflect the normalized reflectance difference (ΔR) of the sample in any two orthogonal directions (such as x and y) within the plane after normal electromagnetic wave incidence, satisfying the equation:

(1) $$\frac{\Delta R}{R}=2\frac{R_{x}-R_{y}}{R_{x}+R_{y}}=2N\left(\theta \right)$$

**FIGURE 3:**
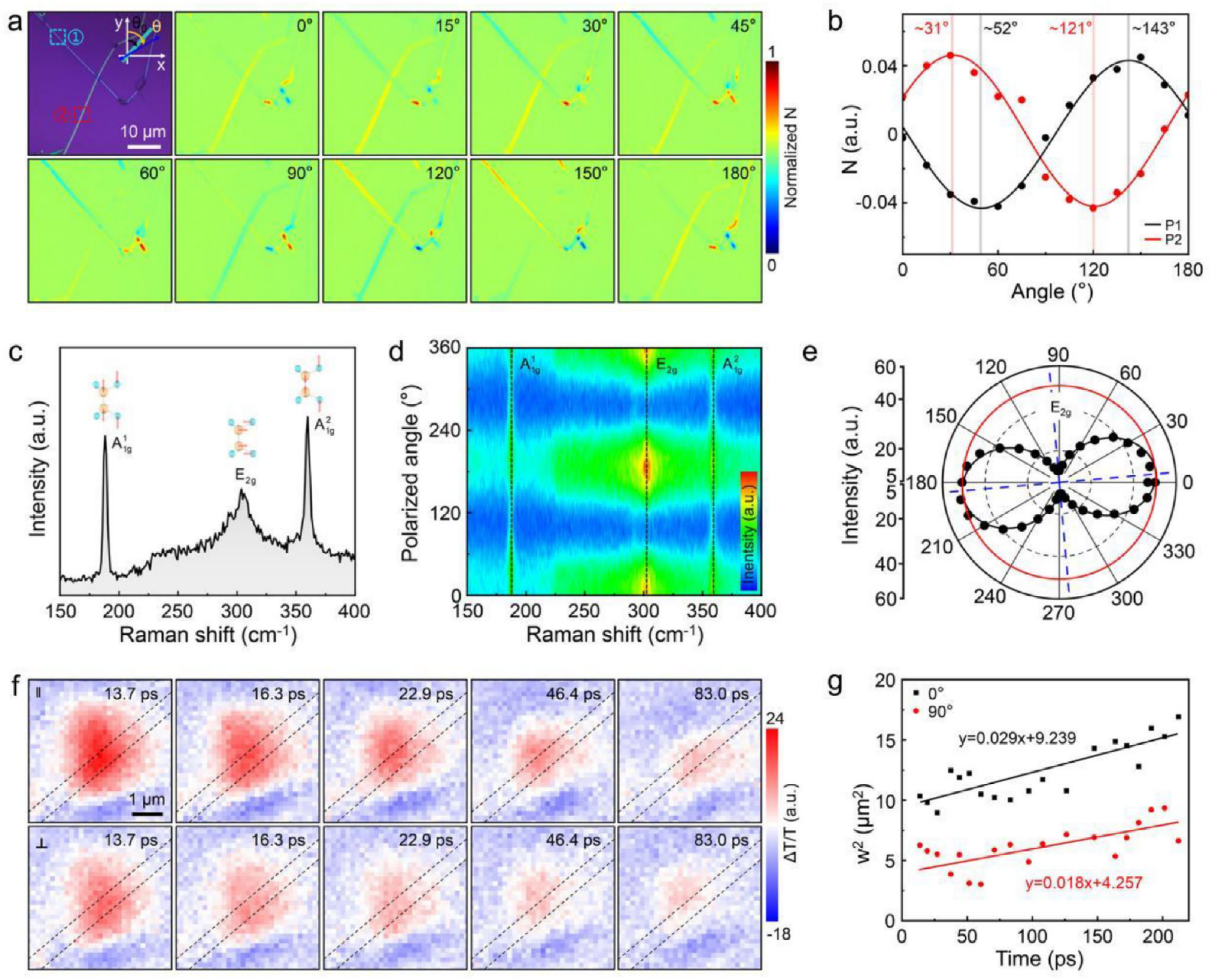
Polarization‐dependent optical characterizations of γ‐GaS nanoribbons. (a) Optical and polarization‐dependent ADRDM images of γ‐GaS nanoribbons. (b) Normalized *N* values of GaS samples in (a), plotted against the incident polarization angle. (c, d) Raman spectrum and polarized contour map of a GaS nanoribbon. (e) Theoretical (red) and experimental (black) polar plots illustrating the polarization‐dependent E_2g_ Raman modes in two‐dimensional (2D) γ‐GaS nanosheets and quasi‐1D nanoribbons. (f) Spatially resolved TA evolution of a GaS sample, highlighting differences in carrier diffusions under polarized excitations. The polarization direction of the excitation light is set to be parallel or perpendicular to the long axis direction of the nanoribbon, which are respectively labeled as “∥” and “⊥”. (g) Time delay‐resolved *ω^2^* extracted from spatial Gaussian fitting in (f).

Where *R_x_* and *R_y_* denote the reflection intensities along the x and y directions, respectively, while the dimensionless value *N*(*θ*) is determined by the equation:

(2) $$N\left(\theta \right)=\frac{2\left(R_{a}-R_{b}\right)}{R_{a}+R_{b}}\cos2\left(\theta -\theta_{0}\right)$$

where *R_a_* and *R_b_* represent the reflectance measured along the a‐ and b‐axes of the GaS nanoribbons, respectively. The definitions of *θ* and *θ*_0_ are the same as those in the model in Figure 2a. Figure 3a displays the ADRMD *N*(*θ*) signal mapping of GaS nanoribbons measured at a fixed wavelength. The background color remains almost unchanged with varying incident light polarizations, indicating no optical discrepancy, while the different color of GaS exhibits significant polarization‐dependent anisotropy [33]. To further elucidate this relationship, we selected two distinct positions (P) on the respective nanoribbons (cyan and red boxes corresponding to P1 and P2, with material thickness and width of ∼110 and 750 nm) and extracted the normalized *N*(*θ*) values followed by fitting using Equation (2). For P1, the minimum *N*(*θ*) value occurred at a polarization angle of approximately 52°, with the maximum at around 143°. For P2, the maximum was at approximately 31° and the minimum at around 121° (Figure 3b). The differential reflection characteristics of the GaS nanoribbons exhibited a standard periodicity of approximately 90° in the form of cosine functions, with the strongest reflection angle consistently corresponding to the material's *a‐axis* direction. This is highly consistent with FEM theoretical predictions and directly validates the optical anisotropy in model analysis. For additional experimental results of GaS materials using the ADRMD technique, refer to Section S7.

Following the material's reflection analysis, we proceeded to investigate its phonon vibrations utilizing Raman spectroscopy, a robust structural characterization technique renowned for its precision in ascertaining the crystal's symmetry [22]. Figure 3c–e presents polarization‐dependent Raman spectra of γ‐GaS nanoribbons under 532 nm laser excitation, revealing three characteristic peaks around 188.3, 303.8, and 358.7 cm^−^^1^, corresponding to the specific modes of $A_{1g}^{1}$, *E*_2*g*_, and $A_{1g}^{2}$ as previous reports [34]. Under the back scattering parallel configuration with fixed samples, all the peaks exhibit polarization dependence. Maximum Raman intensity occurs when polarization aligns with the nanoribbon *b*‐axis (*θ* = 0°), while perpendicular orientation yields minimal intensity (Figure 3e and Section S8). To rigorously validate the correlation, we conducted a quantitative analysis of the Raman intensity (*I_a_*) as follows [22]:

(3) $$I_{a}\propto {\left[e_{i}\cdot R_{a}\cdot e_{s}\right]}^{2}$$

Where the unit polarization vectors of the incident light and the scattered Raman signal are defined as *e_i_* = [cos*θ*, sin*θ*, 0] and *e_s_* = [1, 0, 0]ᵀ, and the Raman tensor of an active mode is *R_a_*.

Since the Raman tensors for *A_1g_* and *E_2g_* modes are

(4) $$A_{g}=\left(\begin{array}{ccc} a & 0 & 0\\ 0 & a & 0\\ 0 & 0 & b \end{array}\right)$$

(5) $$E_{g}\left(x\right)=\left(\begin{array}{ccc} 0 & c & d\\ c & 0 & 0\\ d & 0 & 0 \end{array}\right)$$

(6) $$E_{g}\left(y\right)=\left(\begin{array}{ccc} c & 0 & 0\\ 0 & -c & d\\ 0 & d & 0 \end{array}\right)$$

the Raman intensities can be described as:

(7) $$I\left(A_{g}\right)\propto a^{2}\cdot \cos^{2}\theta$$

(8) $$I\left(E_{g}\left(x\right)\right)\propto c^{2}\cdot \sin^{2}\theta$$

(9) $$I\left(E_{g}\left(y\right)\right)\propto c^{2}\cdot \cos^{2}\theta$$

Notably, the Raman scattering intensity of *A_g_* mode in the experiment aligns well with theoretical predictions, while the *E_g_* mode intensity theoretically ought not to exhibit a spindle‐shaped distribution contingent on the polarization state of the incident light (Figure 3e). This outcome mirrors the alterations induced by the geometric configuration of quasi‐1D GaS again. On a macroscopic scale, polarized light with a polarization direction perpendicular to the nanoribbon demonstrates reduced coupling efficiency with the material. Microscopically, this characteristic may also impose constraints on the phonon vibrations of the material in this orientation.

To further explore the geometry influence microscopically, the transport characteristics of photogenerated carriers of GaS were investigated by using transient absorption (TA) microscopy (detailed in Sections S9 and S10 and Tables S1 and S2) [23, 35]. In the experiment, a 400 nm and 170 fs Gaussian laser pulse with a peak fluence of ∼640 µJ cm^−^² and spot area of 1 µm^2^ was employed to inject electron‐hole pairs in the sample. Then the carrier kinetics were monitored by measuring changes in the differential transmission (ΔT/T) across the 445–540 nm wavelength range and a delay time scale of 0–100 ps following pump excitation. The polarization directions of both pump and probe lights were aligned parallel to the *b‐axis* of the γ‐GaS nanoribbon to maximize the signal‐to‐noise ratio. A pronounced bleach signal corresponding to the material's optical bandgap was observed near 450 nm in the TA spectrum, from which the carrier dynamics of GaS were extracted by analyzing the temporal evolution. Numerical fitting revealed that after an initial transient process lasting about 2 ps, the ΔT/T signal decayed exponentially with a time constant of ∼46 ps. These two characteristic time scales can be attributed to the hot carrier cooling and the interband non‐radiative recombination, respectively [36].

Subsequently, we explored spatial‐dependent carrier diffusion dynamics using a single‐point pump and wide‐field probe configuration, where the pump spot was focused on one point on the sample surface to inject photogenerated carriers, and then the spatial TA signal intensities were recorded by measuring ΔT/T at all probe areas at different delay times (Figure 3f) [35]. As carriers spatially diffuse to ambient positions, the width (*w*, full width at half‐maximum, FWHM) of the diffusion profile can be precisely determined through Gaussian fitting due to the density gradient. The width also broadened with the time delay. Here, the spatial‐dependent ΔT/T intensity distributions were monitored at five specific delay time nodes (13.7, 16.3, 22.9, 46.4, and 83.0 ps). The squared width (*w* ^2^) evolution exhibited a linear relationship with delay time, allowing us to calculate the carrier diffusion constant (*D*) using the carrier diffusion model *w* ^2^(*t*) = *w* ^2^(*t*_0_) +16ln(2)*D*(*t*‐*t*_0_), where *t*_0_ is an arbitrary initial time (Figure 3g). The carrier mobility (*μ*) was then derived using Einstein's relation *μ*/*e* = *D*/*k_B_T*, where *μ*, *e*, *k_B,_* and *T* are the mobility, elementary charge, Boltzmann constant, and temperature, respectively. To assess the anisotropy of GaS, the sample was further rotated 90° clockwise, and the measurements were repeated under identical conditions. It yields room‐temperature diffusion coefficients of 26.1 cm^2^·s^−1^ and 16.2 cm^2^·s^−1^ along and perpendicular to the *b*‐*axis* of γ‐GaS nanoribbon, respectively, along with carrier mobilities of 1010.6 cm^2^·V^−1^·s^−1^ and 627.3 cm^2^·V^−1^·s^−1^. The ratio of the mobilities (*μ_ratio_*) in the two directions is approximately 1.61. These results indicate that photogenerated carriers exhibit more efficient transport along the dominant direction of the nanoribbon, highlighting significant anisotropy influenced by the material's microscopic 1D geometry.

Notably, to ensure the accuracy of the above carrier transport analysis, it is imperative to maintain uniform carrier decay rates across different locations within the carrier distribution profile. To achieve this, we investigate the relationship between the ΔT/T signal and carrier density by varying the pump fluence, ensuring that only the profile height changes without altering its shape or width when the fluence varies. The TA signal remains within its linear regime. This effectively eliminates discrepancies in measurements arising from the material's differential absorption of pumps with varying polarizations. In addition, to minimize the influence of hot carriers on the carrier transport, we select time nodes for measuring the spatial‐dependent ΔT/T intensities that occur after the hot carrier cooling following pump excitation. Finally, it is observed that the ΔT/T intensities can be measured regardless of whether the probe spot is positioned inside or outside the sample. This further underscores that the equivalent in‐plane anisotropy exhibited by our material is an objective reality stemming from its geometry’s effect on light scattering.

The above experimental data, particularly the anisotropic carrier mobility directly observed in the TA experiment, clearly reveal the crucial role of dielectric confinement in the anisotropic behavior of our material. Within the limited subwavelength scale, this confinement leads to an uneven electromagnetic field distribution for different polarization modes propagating inside the γ‐GaS nanoribbon, thereby determining the relationship between material size and the excitation wavelength that yields optimal anisotropy (also supported by Section S6). This is precisely the core message of this work. Nevertheless, we note that within such a confined region, other factors, such as surface roughness, edge scattering, and contact effects, will also become pronounced and collectively influence the material's anisotropy.

### 2.4 Polarization‐Dependent Photoelectric Performance and Applications

**FIGURE 4:**
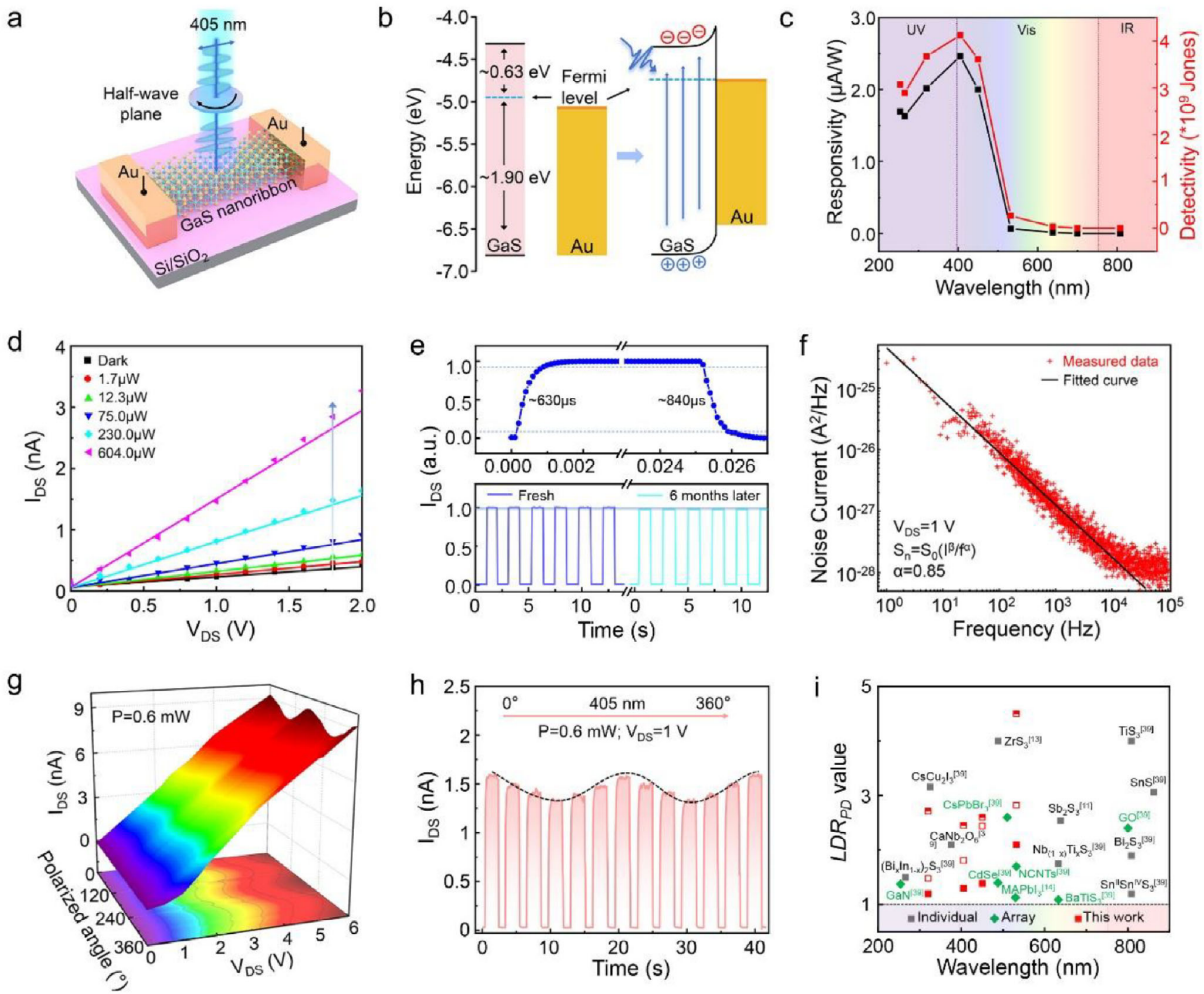
Polarized optoelectronic performance characterizations of γ‐GaS nanoribbon‐based photodetector. (a, b) Energy band theory analysis of GaS photodetector under excitation. Spectral responsivity/detectivity (c), power‐dependent I_DS_‐V_DS_ characteristics (d), normalized time‐resolved photoresponse (e), and noise power spectrum (f) of GaS detectors. (g, h) Polarization‐dependent photoresponse of GaS devices. (i) *LDR*_PD_ values comparison between our GaS device and other 1D material‐based photodetectors. The red hollow, half‐filled, and solid squares represent the theoretical optimum, the unconstrained theoretical optimum, and experimentally measured values, respectively.

Following an in‐depth investigation into the dielectric‐confinement‐induced optical anisotropy in quasi‐1D γ‐GaS nanoribbons, we promptly leverage its inherent properties to broaden its application in polarized photodetection. Experimentally, a single GaS nanoribbon on a SiO_2_/Si substrate (300 nm SiO_2_) was to serve as the conductive channel material. Subsequently, gold electrodes (50 nm thickness) were deposited using ultraviolet (UV) lithography and vacuum deposition techniques, resulting in a double‐ended photodetector configuration (Figure 4a). The nanoribbon exhibited a typical width of ∼750 nm and a thickness of ∼110 nm. Spatial photocurrent scanning imaging revealed that the maximum photocurrent was generated exclusively in the central region of the conductive channel under an applied bias voltage, rather than at the material‐electrode interfaces, suggesting that the photoresponse predominantly relied on the photoconductive mechanism (see Section S11 for details) [37]. Specifically, light exposure induced an increase in photogenerated carriers within the photosensitive material, enhancing device conductivity and enabling a significant photoresponse under an external bias. After determining the material's energy band structure using Kelvin probe force microscopy (KPFM) and UV photoelectron spectroscopy (UPS), the mechanism underlying the photoresponse was simplified as depicted in Figure 4b. Given the optical bandgap of the γ‐GaS nanoribbon (∼2.53 eV), wide‐spectrum measurements demonstrated that the device exhibited a pronounced response, especially around the UV wavelength range (Figure 4c). However, due to intrinsic material defects, longer‐wavelength visible illumination resulted in partial photon capture by the defects, generating an obvious but lower responsivity (*R*). For detailed experimental results, refer to Section S12.

Furthermore, we characterized the photoelectric performance at 405 nm, the wavelength exhibiting the most pronounced photoresponse (∼2.47 µA W^−^^1^, 4.12 × 10^9^ Jones). Figure 4d illustrates the device's current‐voltage (IV) curve in the dark state, revealing a linear relationship that indicates good ohmic contact between the electrodes and GaS material [38]. Then, a photocurrent was generated when the device was exposed to a 405 nm laser. As the light power intensity increased, the response became more pronounced with a power‐law fitting of ∼0.97 at a 1 V bias voltage, suggesting a few impurity defects in the channel layer but excellent photogenerated carrier transport efficiency (Section S12) [38]. Subsequently, we assessed the device's photoresponse speed and observed a rise/fall time of ∼630/840 µs under 1.7 µW laser illumination (Figure 4e). Due to the intact lattice structure and resistance to water and oxygen of GaS, our device demonstrated long‐term environmental stability, retaining over 95% of its initial performance after 6 months of air exposure and 500 testing cycles. Furthermore, we also analyzed the photodetector's noise characteristics, with Figure 4f presenting the noise power spectrum under a 1V bias voltage in the dark state. By fitting the noise power density equation *S* = *S_0_* (*I^β^*/*I^α^*), we determined that the 1/f noise is predominant in the low‐frequency region, with fitting parameters *α* and *β*, and a constant *S_0_* independent of bias. The fitted *α* value of 0.85, close to 1, indicated minimal carrier traps or recombination centers in the photosensitive material and good carrier transport capability (refer to Section S13 for details) [11].

It should be noted that the overall photoresponse performance of our device is moderate, and the key figure of merit, responsivity, is even below average (Table S3). Analysis of the material's light absorption and carrier transport characteristics reveals that low absorption is the fundamental cause of this poor performance, which may further limit the photoelectric conversion efficiency of photogenerated carriers (Section S14). Nevertheless, the photodetectors made of low‐dimensional group III‐VI semiconductors reported to date generally exhibit excellent photoresponse performance, indicating that our γ‐GaS‐based device still holds outstanding potential (Table S4). By optimizing the optical path in detection and improving the lattice quality of the material, the performance of our γ‐GaS device is expected to be further enhanced in the future.

After analyzing the photoelectric performance of GaS photodetectors, we then focused on their polarization‐dependent photoelectric characteristics when they were operated under 1 V bias driving and polarized illumination. The polarization states were precisely controlled via sequential polarization components, including polarizers, half‐wave plates, and Glan‐Taylor prisms. Initially aligned parallel to the *b*‐*axis* of the GaS ribbon, the polarization angle *θ* was systematically varied by rotating the half‐wave plate (Figure 4a). Figure 4g presents a photocurrent map plotted against the polarization states of incident light and the applied bias voltage. Upon extracting photocurrent values at 1 V and varying the polarization angle from 0° to 360°, a wave‐like photocurrent pattern emerged, demonstrating pronounced photoelectric anisotropy (Figure 4h). The data were fitted using the following equation:

(10) $$I_{\mathrm{ph}}\left(\theta \right)\propto I_{\mathrm{ph}-b}\cos^{2}\left(\theta +\varphi \right)+I_{\mathrm{ph}-a}\sin^{2}\left(\theta +\varphi \right)$$

Where *I_ph‐b_* and *I_ph‐a_* are photocurrents along the *b*‐ and *a‐axes* of the crystal, and *φ* represents a fitting parameter. The device achieved a maximum polarization *LDR_PD_* (*I_ph‐b_*/*I_ph‐a_*) of ∼1.3, indicating its capacity to distinguish incident light with different polarization states through geometric modulation of materials. Notably, despite modest photoresponse under 532 nm wavelength laser exposure, our devices demonstrate remarkable polarization sensitivity. The measured *LDR_PD_* value is ∼2.1, which is comparable to detectors based on naturally aligned or artificially oriented 1D materials (Figure 4i) [39, 40, 41, 42, 43, 44, 45, 46, 47, 48, 49, 50, 51]. Moreover, the observed photoelectric anisotropy stems exclusively from the nanoribbons’ 1D geometry rather than intrinsic anisotropic crystallography, also enabling polarization sensitivity across a broad spectral range (see Section S15). The primary advantage of GaS lies in its relatively large and tunable bandgap, which makes it highly promising for applications in optoelectronic devices, particularly in detection across the UV to blue spectral range.

From the above theoretical analysis and experimental observations, a clear gap exists between the simulated *LDR_Abs_* and the measured photoresponse *LDR_PD_*, with the former value consistently larger than the latter. *LDR_Abs_* represents an ideal scenario, where anisotropic light absorption arises from the excitation of various electromagnetic modes within structurally confined materials. However, factors such as inefficient carrier transport, recombination losses, and non‐ideal electrode geometries enhance isotropic carrier scattering in actual measurements, thereby reducing the *LDR_PD_* [39, 40, 41, 42, 43, 44, 45, 46, 47, 48, 49, 50, 51]. The quantitative relationship between *LDR_Abs_* and *LDR_PD_* can be given by:

(11) $$LDR_{\mathrm{PD}}=LDR_{Abs}\cdot \eta_{transport}\cdot \eta_{collection}$$

Where *η_transport_* and *η_collection_* denote the anisotropic retention rates of carrier transport efficiency and collection efficiency within the device, respectively.

To quantitatively analyze this process, we further measured the anisotropic photoresponse of the photodetector under 450 nm laser excitation. Using the carrier mobility anisotropy ratio (*μ_ratio_* = 1.61) obtained above from TA spectroscopy near the 450 nm probe wavelength, we derived *η_transport_* = 1.61/2.44 ≈ 66% and *η_collection_* = 1.39/1.61 ≈ 86%. This indicates that after photogenerated carriers are excited in the material, approximately 34% of the anisotropic weight is lost during carrier diffusion and recombination, while an additional 14% is lost during carrier collection. Notably, although the polarization‐dependent photoconversion efficiency may vary with excitation wavelength, this trend persists, providing guidance for future device performance optimization.

**FIGURE 5:**
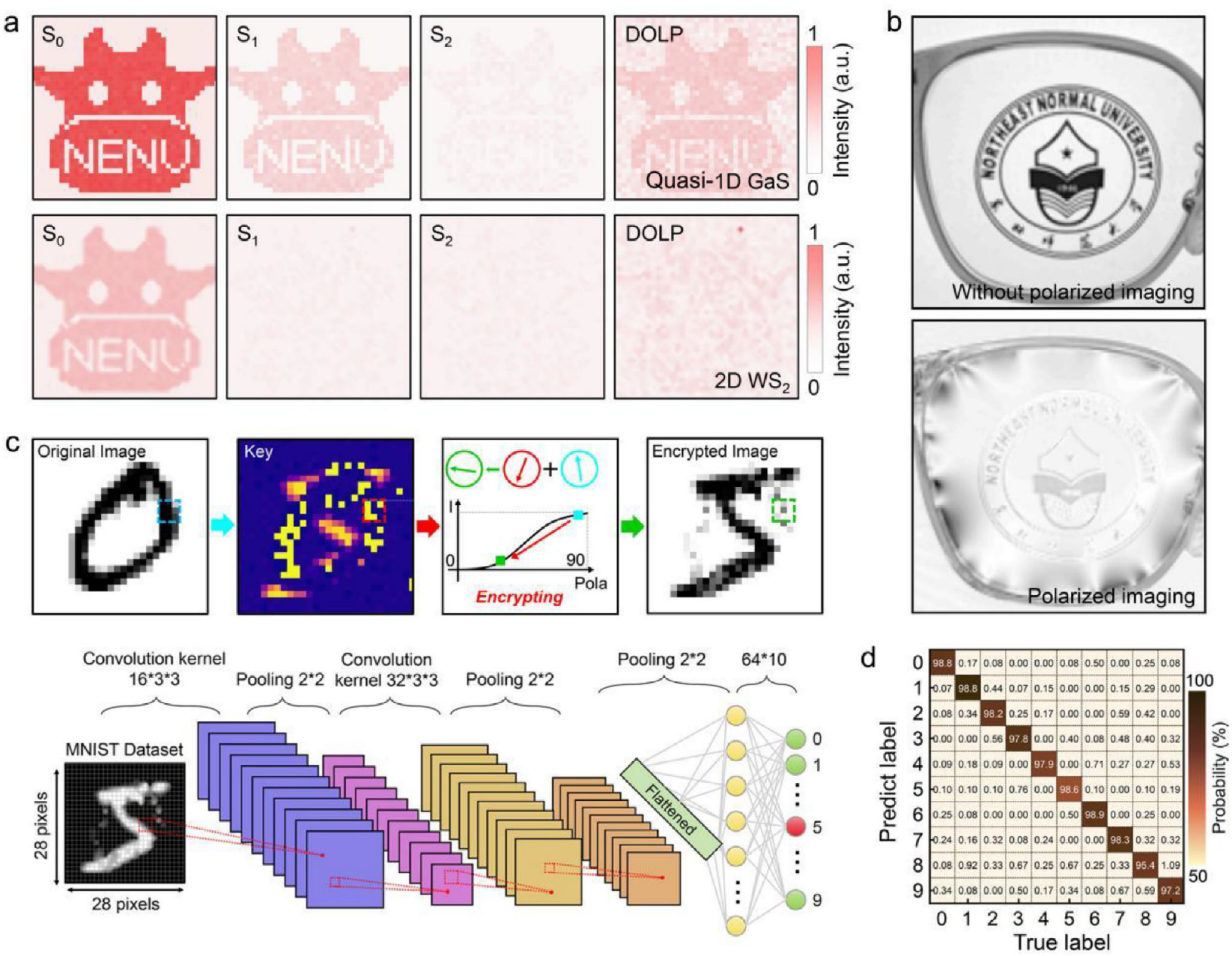
Applications leveraging the polarized photoresponse of GaS detectors. (a) Comparative DOLP imaging patterns of “NENU” using quasi‐1D GaS detectors and 2D WS_2_ devices. (b) Contrast images revealing internal stress distribution in a transparent lens, captured by GaS and WS_2_ photodetectors. (c) Schematic of polarization‐dependent visual cryptography and convolutional neural network architecture. (d) Confusion matrix for image recognition after 40 epochs.

To further evaluate the application potential of our GaS‐based polarized photodetector, we next investigated its performance in polarized imaging, stress detection of transparent materials, and visual cryptography [24, 25, 26]. For polarized imaging demonstration, a hollow aluminum mask (5 × 5 cm^2^) featuring a “cow” pattern and “NENU” letters was positioned between the detector and the 405 nm laser source. The mask's heterogeneous composition (aluminum vs. voids) and rough surface morphology imparted distinct polarization signatures to transmitted light. Experimentally, a collimated beam with a diameter of ∼0.5 cm illuminated the mask while a rotating half‐wave plate modulated the incident polarization state. The detector spatially mapped light intensity across polarization angles (0°, 45°, 90°, and 135°) via point‐by‐point scanning along an “S”‐shaped trajectory perpendicular to the optical axis. By integrating these intensity distributions with Stokes parameters, we reconstructed the mask's polarization information with degree of linear polarization (DOLP), as shown in Figure 5a [6]. For comparison, an isotropic WS_2_‐based photodetector was also fabricated and subjected to identical testing conditions. As expected, it failed to resolve polarization‐dependent contrast (Figure 5a), confirming the critical role of GaS nanoribbons in polarization sensitivity. Detailed experimental protocols for polarization imaging are provided in Section S16.

Leveraging the GaS photodetector's polarization‐sensing capability, we then investigated its application in mapping residual stress distributions within transparent materials, where production‐induced stress alters birefringence and thus modulates transmitted light polarization [25]. When polarized light traverses stress‐varying regions in such materials, spatially dependent birefringence induces phase retardation (Δδ) that correlates with the DOLP and internal stress (Δσ) through the relationship Δδ = arcsin(DOLP) = (2π·C·d·Δσ)/λ, where C denotes the stress‐optic coefficient, d the optical path length, and λ the wavelength [52]. Experimentally, we replaced the aluminum mask with a transparent lens and followed an analogous polarization‐resolved scanning protocol, measuring DOLP spatial distributions to reconstruct the stress maps. As demonstrated in Figure 5b, the GaS detector clearly resolved stress patterns through polarization contrast, whereas the isotropic WS_2_‐based detector failed to produce stress‐discriminative imaging. Detailed methodologies for stress imaging of transparent lenses are provided in Section S17.

Finally, leveraging the polarization‐sensitive photoresponse characteristics of GaS devices, we investigated their potential application in visual cryptography [26]. In the experiment, a polarized light beam with a fixed polarization state was assumed to illuminate uniformly to a transmissive spatial light modulator (SLM), and the transmitted light was subsequently projected onto the GaS detector. The detector also scanned along an “S”‐shaped path perpendicular to the incident light direction, recording photocurrent data to generate a 28 × 28 pixel photocurrent intensity map. By manipulating the rotation of liquid crystal molecules in specific regions of the SLM, the polarization direction of the transmitted light at corresponding positions could be altered. Given that variations in the polarization state induced changes in the GaS detector's output current, rescanning the photocurrent distribution map enabled the retrieval of the initial encoded light intensity pattern (e.g., the number 0 as depicted in Figure 5c). Furthermore, a second SLM was introduced between the first modulator and the detector, and the liquid crystal molecules were similarly controlled to modulate the polarization state of the transmitted light a second time. This stage introduced the spatial‐dependent modifications to the initial pattern, ultimately yielding the encrypted target pattern (e.g., the number 5 in Figure 5c). The key of information encryption and decryption lies in precisely establishing the mapping relationship between each pixel of the spatial image and the operational state of the liquid crystal at the corresponding position on the SLM. Notably, this encryption scheme exploits the correlation between the polarization state of incident light and the photoresponse of the detector, enabling encryption through the diverse manifestations of polarization states.

After completing the above information encryption demonstration, we further employed a convolutional neural network (CNN) to conduct a quantitative analysis of the recognizability and information fidelity of encrypted images. As illustrated in Figure 5c, the network architecture adheres to the classic LeNet‐5 topology, featuring a feature extraction module composed of two sets of alternating convolutional layers (Conv 3 × 3) and maximum pooling layers (MaxPool 2 × 2), followed by two fully connected layers (FC512→FC10) for high‐dimensional feature mapping and classification decision‐making (Method and Section S18). Through 40 rounds of iterative training on the encrypted image dataset, the model achieved a recognition accuracy of 95.56% on the test set, with the cross‐entropy loss converging to 0.16. The dynamic evolution curves of accuracy and loss during the training process are shown in Figures S18b,c. To visually verify the model's generalized recognition capability for various digits, we plotted the normalized confusion matrix for the final training epoch (Figure 5d), revealing that the recognition probability for each digit category exceeded 95%. This indicates that the CNN model exhibits excellent feature decoupling and classification decision‐making capabilities for polarization‐encrypted images.

Traditional optical encryption relies on modulating scalar properties of light such as intensity or phase, and these signals can be directly recorded by standard photodetectors. However, once the encrypted message is intercepted, security depends solely on algorithmic complexity, making it vulnerable to attacks. In contrast, introducing light polarization adds an extra degree of freedom, significantly enhancing information hiding. For example, through spatially varying random polarization modulation, actual images have been demonstrated to be hidden within the polarization distribution [53, 54]. Nevertheless, this process usually relies on a complex optical path for polarization decryption. Polarized photodetectors with intrinsic polarization‐dependent photoresponses are expected to significantly simplify the process. Here, using a γ‐GaS nanoribbon detector as an example, we preliminarily demonstrate its potential for application in this field. With further optimization of device performance, such detectors are expected to significantly enhance the security of optical encryption systems.

## 3 Conclusions

In summary, we synthesized the high‐quality quasi‐1D γ‐GaS nanoribbons via the PVD method. Geometry‐governed dielectric confinement engenders marked differences in near‐field optical scattering, giving rise to an equivalent in‐plane anisotropic response within the isotropic lattice‐structured material. This finding is reinforced by band structure analysis, electric field intensity distribution simulations, and both steady‐state and transient spectroscopy measurements. The universality of this optical anisotropy enables γ‐GaS nanoribbons to exhibit obvious polarization‐dependent photoresponses especially around the UV wavelength range. Under 405 nm laser irradiation, the material demonstrates excellent performances, with a responsivity of 2.47 µA W^−^^1^, a detectivity of 4.12 × 10^9^ Jones, a response time of 630/840 µs, and a *LDR_PD_* value of 1.3. Theoretical forecasts indicate that the *LDR_Abs_* can reach up to ∼4.5 in the 532 nm excitation light band, indicating greater potential for excellent anisotropic photoresponse of our detectors. This remarkable property imparts our devices with application potential in polarized imaging, residual stress detection of transparent materials, and polarization visual cryptography. These applications are difficult to achieve using conventional planar two‐dimensional III‐VI materials. Our work underscores the pivotal role of leveraging geometric dielectric confinements in materials to elicit anisotropic photoresponse, expanding the material toolkit and providing the basis for further enhancing performance of 1D material‐based polarized detectors.

## 4 Method

### 4.1 PVD Growth of Quasi‐1D γ‐GaS Nanoribbons

Quasi‐1D γ‐GaS single‐crystal nanoribbons were synthesized by the traditional PVD method in a horizontal tube furnace (Lindberg, Blue M). 8 mg of Gallium sulfide powder (GaS, 99.99%, Nanjing MKNANO Tech. Co., Ltd., www.mukenano.com) was put at the center of the furnace as precursor, and a SiO_2_/Si plate was placed at ∼8 cm downstream. The furnace was heated from room temperature to 850°C with 80 sccm Ar. Then, 55 sccm Ar and 35 sccm H_2_ were introduced as carrier gas for 7 min. After cooling to room temperature naturally, the quasi‐1D γ‐GaS nanoribbons could be synthesized.

### 4.2 Properties Characterization of γ‐GaS Nanoribbons

The macro/microscopic morphologies of quasi‐1D γ‐GaS nanoribbon were characterized using an optical microscope (OLYMPUS, BX53M), FESEM (Zeiss, Sigma 300), AFM (Bruker, Dimension Icon), and HRTEM (Thermo Fisher, Spectra 300), respectively. The chemical compositions and crystal structure of the material were characterized through XPS (Kratos, AXIS SUPRA+), XRD (Rigaku, Ultima IV), and FFT analysis following surface TEM images. Subsequently, the anisotropic properties of γ‐GaS nanoribbon, including the phonon vibration, carrier dynamics diffusion, and steady‐state reflectance spectrum, were investigated employing an angle‐resolved Raman scattering spectrometer (HORIBA Scientific, LabRAM HR Evolution), a transient absorption spectroscopy system (Time Tech Spectra, TA100), and a homemade ADRDM system, respectively. The KPFM (Bruker, Dimension Icon) and UPS (Thermo Fisher, ESCALAB XI+) were utilized for the energy band alignment analysis of the material.

### 4.3 Optoelectronic Property Theoretical Calculations of Bulk γ‐GaS

The optoelectronic properties of bulk γ‐GaS polytype, crystallizing in the R3m space group with a unit cell containing six Ga and six S atoms, were analyzed through spin‐polarized Density Functional Theory (DFT)‐based first‐principles calculations implemented within the Vienna Ab initio Simulation Package (VASP). The geometry was optimized using the Perdew‐Burke‐Ernzerhof (PBE) functional within the Generalized Gradient Approximation (GGA). To accurately capture the crucial vdW interactions governing the interlayer spacing, the DFT‐D3(BJ) correction was incorporated. The Projector Augmented Wave (PAW) method was used to describe the electron‐ion interactions. The 3d, 4s, and 4p electrons for Ga and the 3s and 3p electrons for S were treated as valence, and the remaining electrons were kept frozen. Both lattice constants and atomic positions were fully relaxed using the conjugate gradient algorithm without symmetry constraints. A plane‐wave energy cutoff of 500 eV and a Monkhorst‐Pack k‐point grid of 21 × 21 × 1 were utilized for both optimization and self‐consistent calculations. The convergence criteria for total energy and interatomic forces were set to 1 × 10^−4^ eV and 0.02 eV/Å, respectively. Based on the optimized structure, we proceeded to investigate the electronic and optical properties. To determine the optical properties, the frequency‐dependent dielectric matrix was calculated after the electronic ground state had been converged. For the dielectric function's broadening, the default CSHIFT value of 0.1 eV was employed, introducing a Lorentzian smearing. The frequency‐dependent complex dielectric function, ε(ω) = ε_1_(ω) + iε_2_(ω), the imaginary part is determined by a summation over empty states using the equation:

(12) $$\begin{array}{ccc} \varepsilon_{2} & = & \frac{4\pi^{2}e^{2}}{\Omega }\underset{q\to 0}{lim}\frac{1}{q^{2}}\sum_{c,v,k}2\omega_{k}\delta \left(\varepsilon_{ck}-\varepsilon_{vk}-\omega \right)\\ & & \times \;\langle \mu_{ck+e_{\beta }q}\left|\mu_{vk}\right.\rangle \langle \mu_{vk+e_{\beta }q}\left|\mu_{vk}\right.\rangle \end{array}$$

Here, the indices c and v refer to conduction and valence band states, respectively, and μ_ck_ is the cell periodic part of the orbitals at the k‐point **k**. The real part of the dielectric tensor ε_1_ is obtained by the usual Kramers‐Kronig transformation.
(13)

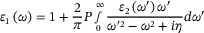

Where P denotes the principal value. The method is explained in detail in the paper by Gajdoš et al. The complex shift η is determined by the parameter CSHIFT. The absorption coefficient can be calculated using the following equation:

(14) $$\alpha \left(\omega \right)=\sqrt{2}{\left[\sqrt{\varepsilon_{1}^{2}\left(\omega \right)+\varepsilon_{2}^{2}\left(\omega \right)}-\varepsilon_{1}\left(\omega \right)\right]}^{\frac{1}{2}}$$

### 4.4 2D‐ACS Simulation of γ‐GaS Nanoribbon under Photoexcitation

The ACS of GaS nanoribbons under illumination varying polarization directions was numerically investigated using a 2D‐FEM method. The simulation structure consists of a GaS nanoribbon embedded in an air environment, with a width (*x*‐*axis*) ranging from 0.5 to 1.5 µm, a height (*z*‐*axis*) between 80 and 160 nm, and an infinite extension along the *y*‐*axis* (Figure 2a). To eliminate boundary reflections, perfectly matched layer (PML) boundaries were implemented along the x‐ and z‐ *axes* of the 2D computational domain. A total‐field scattered‐field (TFSF) source was employed to introduce normally incident linear polarized light along the negative *z*‐*axis* direction across the wavelength range of 300–550 nm, with polarization directions referenced to *y*‐*axis* to analyze polarization dependence. The dielectric constants of GaS within the spectral range were derived from the above‐mentioned first‐principles calculations. The computational mesh adopted a non‐uniform grid configuration, ensuring grid resolution in critical regions remained below 1/20 of the incident wavelength. Convergence validation was performed by iteratively refining the mesh size until the auto‐shutoff criterion reached a minimum threshold of 1×e^−5^, with a material fitting coefficient of 15 and a root‐mean‐square error of 0.00595369. The ACS was determined by monitoring the electromagnetic field distribution around the nanoribbon and integrating the absorbed power via Poynting vector analysis.

### 4.5 Preparation and Performance Measurement of γ‐GaS Polarized Photodetector

A single γ‐GaS nanoribbon was transferred onto a SiO_2_/Si substrate (with 300 nm SiO_2_) using Polydimethylsiloxane (Gel‐pak PDMS). Here, the individual GaS nanoribbon functions as the conductive channel material. PDMS was purchased from Onway Technology Co., Ltd., Shanghai. A metal‐semiconductor‐metal (MSM) photodetector architecture was fabricated through ultraviolet lithography and thermal evaporation of metallic contacts (Au, 50 nm). Photoresponse characterization was then performed using a commercial photoelectric testing system (Metatest, ScanPro Advance) equipped with different laser sources (320, 405, 532, and 638 nm). Linearly polarized illumination was achieved by integrating a Glan‐Taylor prism and an HWP in the optical path, with polarization angles precisely controlled through HWP rotation. For photocurrent imaging, a single‐pixel scanning module synchronized with a motorized stage enabled real‐time photocurrent mapping as the mask plate underwent stepwise translation. Noise spectral density measurements were conducted using a dedicated noise analyzer (Platform Design Automation, FS380 Pro), revealing an ultra‐low noise floor of approximately 10^−28^ A^2^/Hz.

### 4.6 Behavior Model for Pattern Recognition Tasks

The network accepted a single‐channel 28 × 28 input image and processed it through two consecutive blocks, each consisting of a convolutional layer (with ReLU activation), a 2 × 2 max‐pooling layer, and a dropout layer. The first block expanded channel depth to 16 while reducing spatial resolution to 14 × 14, and the second block further increased channel dimensionality to 32 and compressed spatial dimensions to 7 × 7. The resulting feature maps were flattened and passed through a 64‐unit dense layer with dropout, serving as a bottleneck for high‐level representation learning. Finally, a 10‐unit softmax layer produced the predicted class probabilities, and the softmax function converted the raw network outputs into a normalized distribution over the ten digit classes, facilitating straightforward classification decisions. All convolutional and fully connected layers were regularized with an L2 penalty. We trained the model for 40 epochs using the Adam optimizer with a learning rate of 0.0001, balancing convergence stability and computational efficiency.

## Conflicts of Interest

The authors declare no conflicts of interest.

## Supporting information




**Supporting File**: advs75736‐sup‐0001‐SuppMat.docx.

## Data Availability

The data that support the findings of this study are available from the corresponding author upon reasonable request.
