# Supplementary material for: Dielectric‐Confinement‐Induced in‐Plane Photoelectric Anisotropy in Isotropic Quasi‐1D γ‐GaS Nanoribbon
Source: Adv Sci (Weinh). 2026 May 22;13(45):e75736. doi: 10.1002/advs.75736 (PMC13335972; doi:10.1002/advs.75736)
Supplement: Supplementary file 1 [file ADVS-13-e75736-s001.docx]

Supplementary information for

**Dielectric-Confinement-Induced In-Plane Photoelectric Anisotropy in Isotropic Quasi-1D γ-GaS Nanoribbon**

Jiawei Jing^1,#^, Jiamei Chen^1,#^, Xing Xin^1,#^, Peisong Peng^2^, Zhihao Wu^2^, Weiheng Zhong^1^, Hanyu Zhang^1^, Wanfu Shen^3^, Jihua Zhang^4^, Yuwei Shan^5^, Yuanzheng Li^1^, Xingang Zhao^1^, Wei Xin^1,*^, Fang Wang^2,*^, Weida Hu^2^, Haiyang Xu^1,*^, Yichun Liu^1^

1 State Key Laboratory of Integrated Optoelectronics, Key Laboratory of UV-Emitting Materials and Technology of Ministry of Education, Northeast Normal University, Changchun 130024, China

2 State Key Laboratory of Infrared Physics, Shanghai Institute of Technical Physics, Chinese Academy of Sciences, Shanghai 200083, China

3 State Key Laboratory of Precision Measuring Technology and Instruments, School of Precision Instrument and Optoelectronics Engineering, Tianjin University, Tianjin 300072, China

4 Songshan Lake Materials Laboratory, Dongguan, Guangdong 523808, China

5 State Key Laboratory of Luminescence Science and Technology, Chinese Academy of Sciences, Changchun Institute of Optics, Fine Mechanics and Physics, Changchun 130033, China

^#^These authors contributed equally.

*Email: xinwei@nenu.edu.cn, fwang@mail.sitp.ac.cn and hyxu@nenu.edu.cn

Keywords: quasi-1D γ-GaS nanoribbon, isotropic materials, geometry-governed dielectric confinement, equivalent in-plane anisotropy, polarized photodetection

**Supporting Information S1**

**Growth diversity of γ-GaS nanoribbons**

**Figure S1.** (a-b) Optical microscopy (OM) images of quasi-1D γ-GaS nanoribbons. (a) The samples grown at different temperatures with a fixed growth time of 8 minutes and (b) grown at 850 °C with different growth times. Scale bar: 5 μm. (c) Atomic force microscopy (AFM) images of the representative γ-GaS nanoribbons with different thicknesses. White dashed lines mark the scanning paths for sample height profile measurements, revealing thicknesses of about 87.4, 120.1, 93.4, 64.6, and 139.7 nm. Scale bars: 1 μm. Statistical analysis of the (d) thickness and (e) width distribution of γ-GaS nanoribbons, based on a study of nearly 1300 individual nanoribbons.

We systematically explored morphology evolution of γ-GaS nanoribbons with growth temperature and duration in Figure S1a, b. As shown in Figure S1a, the thickness of γ-GaS nanoribbons gradually increases as the temperature increases from 800 to 850 ℃, approximately from 80 nm to 160 nm. With further increasing temperature to 870 ℃, the thickness of γ-GaS nanoribbons stabilized and two-dimensional (2D) deposits appeared. Maintaining a constant growth temperature and increasing the growth duration contribute to an increase in the width of the nanoribbons, ranging from 500 nm to 1500 nm. Substantial experimental data and statistical analyses (Figure S1d, e) show that the widths and thicknesses of γ-GaS nanoribbons are concentrated, with widths around 750 nm and thicknesses near 110 nm.

**Supporting Information S2**

**Synthesis stability determination of γ-GaS nanoribbons**

Figure S2. Optical microscope images of GaS nanomaterials grown at (a) 850 ℃, (b) 860 ℃ and (c) 870 ℃. (d-e) Cross-sectional scanning transmission electron microscopy (STEM) image of the 2D deposits (d) and GaS nanoribbons (e), respectively corresponds the region in blue and red boxes in Figure S2c. The elemental intensity distribution profile extracted along the purple and orange dashed line, respectively. (f) The side-view schematic diagram of β-GaS and γ-GaS.

At growth temperatures maintained within 800-850 °C, γ-GaS nanoribbons nucleate and grow, with their thickness increasing progressively as the temperature rises (Figure S2a). When the temperature is elevated to 860 °C, 2D deposits emerge on the substrate surface. Upon further heating to 870 °C, the thickness of these deposits increases further (Figure S2b, c). Cross-sectional scanning transmission electron microscopy (STEM) was employed to characterize the phase of the 2D deposits. As shown in Figure S2d, their atomic stacking configuration matches that of the β-GaS structure (Figure S2f). Notably, the cross-sectional STEM image of the GaS nanoribbons grown at 870 °C (Figure S2e) still retains the structural characteristics of γ-GaS (Figure S2f). This result demonstrates that the formation of the β-GaS phase at high temperatures does not alter the phase of the nanoribbons themselves, which remain in a highly pure γ-phase. By confining the growth temperature within the optimized stable range of 810-850 °C, the controlled synthesis of high-phase-purity γ-GaS nanoribbons can be realized.

**Supporting Information S3**

**Lattice structure analysis of multilayer γ-GaS nanoribbons**

**Figure S3.** (a) Comparison between the XRD spectra of γ-GaS samples with the standard data. (b) Schematic γ-GaS crystal structures as viewed along different coordinate axes. (c,d) HRTEM top-view images of surface and edge of GaS nanoribbons.

The weak diffraction peaks at 2θ = 21.0° and 23.3° in the X-ray diffraction (XRD) pattern stem from signals of the exposed substrates (Figure S3a). Since the quasi-one-dimensional (quasi-1D) γ-GaS nanoribbons do not completely cover the substrate, the test spectrum inevitably contain the signal from substrate material. Cross-referencing with the International Centre for Diffraction Data (ICDD) Powder Diffraction File (PDF) database (PDF#25-0344, PDF#42-1278) indicates that these two weak peaks correspond to trace Ga-rich or S-rich species residual from the decomposition of GaS precursors during the high-temperature physical vapor deposition (PVD) process. However, the integrated intensity ratio of each peak relative to the dominant γ-GaS (006) peak is less than 5%, confirming the high phase purity of the sample. In the subsequent fabrication of detectors, the target γ-GaS nanoribbons were transferred to a fresh substrate via the proven dry transfer technique, which eliminates interference from residual substances effectively.

**Supporting Information S4**

**First-principles calculations of γ-GaS bulk material**

**Figure S4.** (a) Integral path of the band structure. (b) Calculated absorption coefficient (α) along the a-, b- and c-axes of the bulk γ-GaS. (c) Partial charge density of γ-GaS at the state of CBM and VBM.

**Supporting Information S5**

**Photoluminescence (PL) spectrum of γ-GaS nanoribbon**

**Figure S5.** PL spectrum of γ-GaS under 325 nm excitation. Due to the indirect bandgap of multilayered GaS material, a modest PL peak appears at around 489.4 nm, corresponding to the optical bandgap of ~2.53 eV.

**Supporting Information S6**

**2D ACS simulations of γ-GaS nanoribbons**

**Figure S6.** Anisotropic near-field electric field intensity distributions and ACS in x-z-plane of γ-GaS nanoribbons under (a) 320 nm, (b) 450nm and (c) 532 nm light illumination with s- and p-polarizations. (d) Simulated anisotropic absorption cross-section ratio (*LDR_Abs_*) values as the w/t ratio tends to infinity. (e) Scanning spectra of *LDR_Abs_* for a material with thickness of ~110 nm and width of 750 nm.

Despite γ-GaS’s symmetric crystal structure, its quasi-1D geometry endows the nanoribbons with equivalent in-plane optoelectronic anisotropy. Macroscopically, the nanoribbon’s absorption varies with the angle between the light polarization direction and the ribbon’s b-axis. We employed the finite element method (FEM) to simulate Anisotropic near-field electric field intensity distributions and ACS (Figure S6). Optical parameters for γ-GaS were derived from the first-principles calculations in main text, while those for the SiO₂/Si substrate were sourced from Reference 1. Given that the length of the GaS nanoribbon significantly surpasses its cross-sectional dimensions, periodic boundary conditions were imposed along the y-axis to simulate an infinite structure. Perfectly matched layer (PML) boundary conditions were applied along the x- and z-axes. Then, a plane wave source with varying polarization angles *θ* normally irradiated the ribbon along negative z-axis. We defined the simulation parameters as the ribbon’s height and width. Based on SEM and AFM results, the height range was set to 80-160 nm, and the width range to 0.5-1.5 μm. The wavelengths of the polarized light were set as 320 nm, 405 nm, and 532 nm, respectively (Figure 2).

The ACS, defined as the material’s absorption capacity in the x-z-plane per unit length along y axis, yielded values of 2.52×10^-10^ m for s-polarized and 1.70×10^-10^ m for p-polarized illumination, with an anisotropy ratio (called as differential ACS) of ~1.48. Yielded values of 3.19×10^-10^ m for s-polarized and 1.31×10^-10^ m for p-polarized illumination, with an anisotropy ratio (called as differential ACS) of ~2.44 at 450 nm. Yielded values of 2.78×10^-10^ m for s-polarized and 9.87×10^-11^ m for p-polarized illumination, with an anisotropy ratio (called as differential ACS) of ~2.82 at 532 nm.

To verify the reliability of the model, we calculated the *LDR_Abs_* values as the w/t ratio tend to infinity (Fig S6d). The results show that across the 200–2000 nm spectral range, *LDR_Abs_* converges to 1.000-1.002, indicating the vanishing of anisotropy once the geometric boundary constraints are removed. This result is consistent with the theoretical expectation for an in-plane isotropic two-dimensional material at infinite lateral extent.

The optical anisotropy of nanoribbons is closely related to factors such as material dimensions, incident wavelength, and electromagnetic coupling within specific structural models. Keeping the geometric parameters of γ-GaS nanoribbons fixed, we measured the variation of the *LDR_Abs_* as a function of excitation wavelength. As the wavelength increases monotonically from 300 nm to 600 nm, the *LDR_Abs_* value consistently deviates from unity and exhibits fluctuations (Fig S6e). This behavior confirms the dielectric confinement mechanism, which generally gives rise to anisotropy. Moreover, because different wavelengths can excite distinct resonant modes inside the nanoribbons, the *LDR_Abs_* value inherently varies with the wavelength.

**Supporting Information S7**

**Azimuth-dependent reflectance difference microscopy of γ-GaS nanoribbons**

**Figure S7.** (a) Schematic of azimuth-dependent reflectance difference microscopy (ADRDM) based on a liquid crystal variable retarder (LCVR). (b) Reflectance difference (*N*) spectra of γ-GaS nanoribbon. (c) ADRDM images of GaS nanoribbons at 600 nm laser excitation with polarization state *θ* changing from 0° to 180° (step size: 30°). (d) Dimensionless *N*(*θ*) of GaS sample marked in a box in figure S7 (c), fitting curve extracted from ADRDM.

Materials with asymmetric lattices usually exhibit anisotropic optical responses in broad range. To reveal the equivalent in-plane anisotropy of quasi-1D γ-GaS ribbon, here we conducted the differential reflection spectrum measurement within 500-900 nm wavelength range by using the ADRDM technique (Figure S7a).^2^ To assess the anisotropy of the nanoribbons, we employed ADRDM imaging under a 600 nm laser, where the optical anisotropy peaked within the tested wavelength range (Figure S7b). Changes in the incident light’s polarization states yielded corresponding variations in the *N* values in the nanoribbon region (Figure S7c, d), directly confirming the in-plane optical anisotropy. It is important to note that instrumental constraints limited our measurements to wavelengths above 500 nm. However, this did not preclude us from concluding that the samples exhibit anisotropy in a broad spectral range. Additionally, the tested sample had a thickness/width of ~110/750 nm. According to the simulations presented in the main text, the anisotropy varies with geometric structures, which also explains why our results demonstrated the strongest anisotropic response near the 600 nm wavelength.

**Supporting Information S8**

**Angle-resolved polarized Raman spectroscopy of γ-GaS samples**

**Figure S8.** Polar plots of angle-resolved peak intensities of the $A_{1g}^{1}$(188.3 cm^-1^) and $A_{1g}^{2}$ (358.7 cm^-^¹) Raman modes.

**Supporting Information S9**

**Transient absorption spectroscopy measurement of γ-GaS nanoribbons**

**Figure S9.** (a) 2D color plot of TA spectra of γ-GaS ribbons. (b) TA kinetics (ΔT/T) of γ-GaS samples measured with a 400 nm laser pump and ~450/468/487/513 nm probes. The solid line indicates a two exponential fit with a time constant of τ_eff_. (c) Spatial profile evolution of ΔT/T signals pumped at the center of the GaS nanoribbons, obtained with probe delays of 13.7, 21.6, 30.1, 41.7 and 54.4 ps, respectively. The solid lines are Gaussian fits. The left and right results are measured when the polarization state of pump is parallel and perpendicular to the b-axis of the ribbons.

We employed microscopic TA spectroscopy (ΔT/T) to investigate the kinetics of photogenerated carriers in γ-GaS nanoribbons following photoexcitation. Under 400 nm pump light excitation at a power density of 640 μJ/cm, ΔT/T signal of the ribbons was measured across 445-540 nm wavelength range (Figure S9a). Due to a thin-film interference-like effect (material thickness of ~110 nm), multiple bleaching signals were observed around 450 nm, 468.3 nm, 486.5 nm, and 512.5 nm. Fitting the carrier dynamics at these wavelengths using a double-exponential decay yielded effective carrier lifetimes (*τ_eff_*) of about 41.3 ps (Figure S9b, Table S1). The *τ_eff_* were obtained from:

$\frac{\Delta T}{T}=A_{1}e^{-t/\tau_{1}}\text{+}A_{2}e^{-t/\tau_{2}}\text{+C}$ (1)

$\tau_{\text{eff}}=\frac{A_{1}\tau_{1}^{2}+A_{2}\tau_{2}^{2}}{A_{1}\tau_{1}+A_{2}\tau_{2}}$ (2)

Where the *τ_1_* and *τ_2_* represent time constants, and *A_1_* and *A_2_* are weighting factors. Subsequently, we utilized a single-point pump and wide-field probe configuration to map the ΔT/T signals across the detection area, enabling spatial analysis of carrier diffusion along the a-axis and b-axis under different polarized light excitation (Figure S9c). The probe wavelength was set at 450 ± 20 nm. By extracting spatially resolved ΔT/T data at different pump-probe delay times and performing Gaussian fitting, we obtained the full width at half maximum parameters for each delay. Einstein’s relation fitting of these parameters allowed us to determine carrier mobilities along different axes of GaS nanoribbons (Figure 3g).

**Table S1.** Effective lifetime fitting parameters of a γ-GaS nanoribbon

| Wavelength | A_1_ | τ_1_ (ps) | A_2_ | τ_2_ (ps) | τ_eff_ (ps) |
| --- | --- | --- | --- | --- | --- |
| 468.3 nm | 1.18012 | 2.23765 | 0.2534 | 53.66433 | 45.30173 |
| 486.5 nm | 0.99835 | 1.43194 | 0.50588 | 39.42862 | 36.88746 |
| 512.5 nm | 1.00549 | 1.6791 | 0.57327 | 44.54325 | 41.88496 |

**Supporting Information S10**

**TA spectroscopy of another γ-GaS nanoribbon**

**Figure S10.** (a) 2D TA spectra and (b) ΔT/T signals of γ-GaS nanoribbons.

To further confirm that the bleaching signals observed at various wavelengths stem from the interference effects at γ-GaS material interface, we performed the 2D TA spectra on another sample with a distinct thickness (Figure S10). Bleaching were also observed at wavelengths of about 452.1, 472.7, 497.3, and 523.9 nm for this sample. Given its greater thickness compared to the sample in Figure S9, the strongest bleaching signal exhibited a red-shifted wavelength, consistent with expectations. By applying a double-exponential fit to the dynamics curve of the sample, we determined an effective carrier lifetime of approximately 16 ps (Table S2).

**Table S2.** Effective lifetime fitting parameters of another γ-GaS nanoribbon

| Wavelength | A_1_ | τ_1_ (ps) | A_2_ | τ_2_ (ps) | τ_eff_ (ps) |
| --- | --- | --- | --- | --- | --- |
| 452.1 nm | 0.72629 | 1.35394 | 0.6883 | 17.15987 | 15.94507 |
| 472.7 nm | 0.64132 | 3.35102 | 0.29423 | 20.89944 | 16.35480 |
| 497.3 nm | 0.52323 | 14.54416 | 0.52323 | 17.77619 | 16.32178 |
| 523.9 nm | 0.35362 | 5.31007 | 0.37425 | 18.05073 | 15.27961 |

**Supporting Information S11**

**Band determination of γ-GaS ribbon and its photoresponse mechanism analysis**

**Figure S11.** (a) Kelvin probe force microscopy (KPFM) image of the γ-GaS detector, with a red dotted line marking the scanning path for surface potential measurement. The contour map reveals a potential difference of ~75 mV between γ-GaS and Au electrode. (b) Ultraviolet photo-electron spectroscopy (UPS) of GaS material. (c) Scanning photocurrent image of GaS detector, excited under a 405 nm laser wavelength and a 1 V bias. The insert is a scanning photocurrent image of the device at 0 V bias.

The band alignment of different materials within γ-GaS detector was examined by using the UPS measurements, with the valence band designated as zero potential energy reference. The Fermi level offset of each material relative to its valence band was determined from the slope of the curve’s terminal region in Figure S11b, yielding a value of ~1.37 eV for γ-GaS. The work function of γ-GaS was further calculated by subtracting the energy corresponding to the slope’s extension from the left region of the curve (16.25 eV), using the photon energy (21.22 eV) of the helium I light source, resulting in a work function of -4.97 eV and a valence band potential energy of -6.86 eV. PL measurements in Figure S5 indicated a bandgap of 2.53 eV for γ-GaS, allowing the conduction band potential energy to be derived as the sum of the valence band potential and the bandgap, giving -4.33 eV. The resulting band structure, plotted based on these experimental data, is presented in Figure 4b of the main text.

**Supporting Information S12**

**Photoelectrical performance of γ-GaS photodetectors**

**Figure S12.** (a) The fitting curve of photocurrent versus power density of a γ-GaS detector under 405 nm laser irradiation. Power-dependent I_DS_-V_DS_ curves under (b) 320 nm and (c) 532 nm laser irradiation.

**Supporting Information S13**

**Noise analysis of γ-GaS photodetectors**

**Figure S13.** Noise power spectrum of the γ-GaS detector under V_DS_ = -1, 2 and -2 V bias.

**Supporting Information S14**

**Performance comparison between our γ-GaS photodetector with other low-dimensional (LD) photodetectors**

**Figure S14**. (a) Absorption spectra of single γ-GaS nanoribbon and monolayer Graphene. (b) OM image of a γ-GaS photodetector based on a dense nanoribbon network. (c) Photoresponse of the network device under 405 nm laser excitation.

A performance comparison between our GaS photodetectors with other nanowire /nanoribbon devices is summarized in Table S3, mainly focusing on responsivity and response speed. Our single‑ribbon device exhibits a modest responsivity (~3 μA/W) but a relatively fast rise/fall time of ~630/840 μs. This trade‑off is consistent with the fundamental inverse relationship between photoconductive gain and response speed. The faster carrier recombination and photoresponse leads to lower gain generally.

To identify the origin of the modest responsivity, both the carrier lifetime and the light absorption of single γ‑GaS nanoribbon were measured. Transient absorption (TA) spectroscopy reveals an intrinsic carrier lifetime of ~41.3 ps, which is similar to that of typical 2D transition metal dichalcogenides (e.g., MoS_2_, WS_2_), ruling out ultrafast non‑radiative recombination as the limiting factor.^3^ Instead, absorption measurements (Fig S14a) show that the absolute light absorption of the single nanoribbon is even lower than that of monolayer graphene of ~2.3%, due to the severe spatial mismatch between its sub‑wavelength cross‑section and the micron‑scale laser spot.^4^ This weak light‑matter interaction is the main cause of the poor responsivity. This conclusion is further supported by the performance of a network device (Fig S14b), which exhibits a three‑order‑of‑magnitude increase in responsivity to ~1.56 mA/W, reaching values comparable to those of other LD photodetectors (Fig S14c).

Despite the modest performance of our single‑ribbon device, a broader statistical survey of LD III‑VI group semiconductor photodetectors reveals that these materials typically exhibit high responsivity, ranging from mA/W to A/W. This indicates that γ‑GaS itself possesses intrinsic photosensitivity. Therefore, with further improvements in material quality and optimization of optical coupling strategies, the performance of our devices is expected to be significantly enhanced (Table S4).

**Table S3.** Performance comparison of different nanowire /nanoribbon photodetectors

| Type | Material | Response time (s) | Responsivity (A/W) | Bias (V) | Ref. |
| --- | --- | --- | --- | --- | --- |
| Self-powered | ZrGeTe_4_ | 4×10^-3^ | 6.25×10^-1^ @1064nm | 0 | [5] |
|  | Nb_2_Pd_3_Se_8_ | 5.5×10^-2^/  5.0×10^-2^ | 2.74×10^-3^ @532nm | 0 | [6] |
|  | MoTe_2_ | 3.1-4.3×  10^-5^ | 4×10^-4^ @532nm | 0 | [7] |
|  | NdSb_2_ | 1.5×10^-5^ | 4.9×10^-4^ @532nm | 0 | [8] |
|  | ReS_2_ | —— | 1.5×10^-6^ @532nm | 0 | [9] |
| Under bias | ZrS_3_ | —— | 2.3×10^-1^ @520nm | 3 | [10] |
|  | Sb_2_S_3_ | 4.7×10^-4^ / 6.8×10^-4^ | 3.434×10^-1^ @450nm | 2 | [11] |
|  | CrPS₄ | ＜2 | 1.37×10^-7^ @405nm | 5 | [12] |
|  | Ga_2_O_3_ | 8.6×10^-4^ | 2.3×10^-1^ @262nm | 5 | [13] |
|  | BP | 4×10^-5^ | 3.5×10^-3^ @1200nm | 0.1 | [14] |
|  | CdSe | —— | 3×10^-1^ @400nm | 35 | [15] |
|  | InP | —— | 9.6×10^-1^ @740nm | 3 | [16] |
|  | Si | 4.6×10^-2^/  3.7×10^-2^ | 2.458×10^-2^ @980nm | -1 | [17] |
|  | Ta_2_NiS_5_ | 3.1×10^-5^ | 2.5×10^-3^ @632nm | 1 | [18] |
|  | Nb_2_Pd_0.71_Se_5_ | 8.18×10^-6^/  8.65×10^-6^ | 1 @638nm | 0.1 | [19] |
|  | ZnS | 3×10^-1^ | 1.2×10^-1^ @320nm | 5 | [20] |
|  | ZnSe | 3×10^-1^ | 1.2×10^-1^ @400nm | 30 | [21] |
|  | HfS_3_ | 2×10^-1^ | 6×10^-5^ @500nm | 5 | [22] |
|  | ZrSe_3_ | 4×10^-1^ | 5.3×10^-1^ @650nm | 5 | [23] |
|  | HfSe_3_ | 4×10^-1^ | 1.2×10^-2^ @532nm | 5 | [23] |
|  | This work | 6.3×10^-4^/  8.4×10^-4^ | 2.47×10^-6^ @405nm | 1 | — |

**Table S4.** Performance comparison of different LD III‑VI group semiconductor photodetectors

| Material | Response time (s) | Responsivity (A/W) | Bias (V) | Ref. |
| --- | --- | --- | --- | --- |
| GaS | ＜3×10^-2^ | 9.5×10^-1^ @550nm  1.56 @490nm  1.53×10^1^ @365nm  1.92×10^1^ @254nm | 2 | [24] |
| GaS | ~1×10^-2^ | 6.443×10^1^ @633nm | 1 | [25] |
| InSe | 1×10^-3^/10×10^-3^ | 1×10^5^ @633nm | 2 | [26] |
| GaS | —— | 1.78×10^-1^ @405nm | 5 | [27] |
| GaS | —— | 1.77×10^-2^ @405nm | 5 | [27] |
| GaS | 1.21×10^-5^/1.29×10^-5^ | 4.6×10^-3^ @380nm | 0 | [28] |
| GaS | 1.29×10^-5^/1.3×10^-5^ | 1.1×10^-2^ @380nm | 0 | [28] |
| InSe | 7×10^-3^ | 3.3~4.9×10^-6^ @sunlight | 0-1.0 | [29] |
| GaS | —— | 4.77×10^-3^ @300-800nm | 0.6 | [30] |
| GaS | —— | 6.8×10^-3^ @275nm | 1.1 | [31] |
| InSe | 5×10^-2^ | 3.9×10^-3^ @633nm  1.23×10^-2^ @450nm | —— | [32] |
| InSe | 5×10^-3^/8×10^-3^ | 3.06×10^4^ @700nm | 5 | [33] |
| GaSe | 2.7×10^-4^ | 5×10^3^ @410nm | 8 | [34] |
| GaTe | 6×10^-3^ | 1×10^4^ @532nm | 5 | [35] |
| InSe | 2×10^-2^ | 1.7×10^4^ @700nm | 1 | [36] |
| GaS | 6.6×10^-2^ | 4.7 @275nm | 20 | [37] |
| GaSe | —— | 1.7×10^-2^ @405nm | 10 | [38] |
| GaSe | 6×10^-3^/10×10^-3^ | 2.7×10^-3^ @532nm | 5 | [39] |
| InSe | 6.2×10^-4^/ 5.4×10^-4^ | 1.94×10^2^ @800nm | 1 | [40] |
| GaSe | —— | 1.7 @400-800nm | -10 | [41] |
| GaSe | 2×10^-2^ | 2.8 @254nm | 5 | [42] |
| GaS | 3×10^-6^/ 8.3×10^-6^ | ~1.73×10^3^ @405nm | 2 | [43] |

**Supporting Information S15**

**Polarization-dependent photocurrent measurements of γ-GaS photodetectors**

**Figure S15**. (a-d) Polarization-sensitive photocurrent measurements of γ-GaS photodetectors under laser excitation with 320, 405, 450 and 532 nm wavelengths, respectively, with a consistent bias voltage of 1 V applied. (e) Polarized photocurrent imaging of a γ-GaS device, conducted under a 405 nm laser wavelength and a 1 V bias voltage.

The theoretically computed anisotropy ratio of the absorption cross-section defines the physical upper limit for a photonic structure, representing the ideal scenario of perfect photon-to-carrier conversion. In practice, however, the measured photocurrent polarization ratio is invariably lower, as it is governed by the entire photoconversion chain—from initial light absorption to ultimate charge collection by electrodes. This process is susceptible to a multitude of non-ideal factors, including intrinsic recombination losses, inefficient carrier transport, and non-ideal electrode geometries. ^44-46^ Consequently, the observed discrepancy, wherein the theoretical absorption polarization ratio exceeds its experimental photocurrent counterpart, is a ubiquitous and expected phenomenon in nanophotonic devices.

To establish the quantitative relationship between simulated anisotropic absorption cross-section ratio (*LDR_Abs_*) and linear dichroism ratio of photoresponse (*LDR_PD_*), we derive the physical relationship based on Ohm’s law as follows:^47,48^

$J=\sigma E$ (3)

$I_{\text{ph}}\text{=}q\frac{P}{h\upsilon}\cdot EQE=\eta\cdot G\cdot q\mu\tau E\cdot S=C\cdot G\cdot\mu\tau$ (4)

Based on $\text{I(x)}=I_{0}e^{-\alpha x}$, the following formula can be derived:

$G\approx\eta\cdot\frac{\alpha P}{h\upsilon A}$ (5)

So $I_{\text{ph}}\text{=}\frac{\alpha}{h\upsilon}\cdot\eta\cdot P_{0}\cdot q\mu\tau E$，where *α* is absorption coefficient, *E* is photon energy, *η* is quantum efficiency, *P_0_* is incident light power, *μ* is carrier mobility, τ is carrier lifetime, *E* is electric field strength, and G is photoresponse gain of the γ-GaS device.

Then, the relationship between *LDR_Abs_* (*α_ph-b_*/*α_ph-a_*) and *LDR_PD_* (*I_ph-b_*/*I_ph-a_*) can be derived from the above model by introducing the anisotropic efficiency factors:

$LDR_{photo}=LDR_{abs}\cdot\eta_{transport}\cdot\eta_{collection}$ (6)

where η*_transport_* and η*_collection_* denote the anisotropic retention rates of carrier transport efficiency and collection efficiency within the device, respectively.

In the photoresponse experiments, 405 nm laser excitation was employed to evaluate the optimal overall performance of the device—including high responsivity, an excellent anisotropy ratio, and fast response speed—making this wavelength suitable for assessing practical application potential. For transient absorption (TA) measurements, 450 nm was selected as the probe wavelength because its photon energy (~2.76 eV) lies close to the optical bandgap of γ-GaS, where the bleaching signal arising from carrier transitions is most pronounced and effectively reveals the anisotropic characteristics of carrier transport.^49^ Performing TA measurements under 405 nm excitation would populate higher-energy states, where rapid thermal relaxation obscures the bleaching signal. This hinders the accurate extraction of kinetic parameters of carrier diffusion, which prevents a meaningful anisotropy analysis.

It should be noted that although the excitation wavelength used for the γ-GaS detector differs from the optimal probe wavelength in the TA measurements, both reside within the same conceptual framework of dielectric-confinement-induced optical anisotropy in geometrically confined materials. Consequently, the findings reveal consistent physical principles. To further substantiate this, we performed additional polarization-dependent photoresponse measurements under 450 nm laser excitation (Fig S15d). Although the wavelengths used in the two experiments differed, these data do not alter the core conclusions of this work. The dielectric-confinement-induced anisotropy originates from the macroscopic modulation of the local electromagnetic field distribution by the material's microstructure and is universal across a broad spectral range.

**Supporting Information S16**

**System configuration of γ-GaS polarized imaging system**

**Figure S16.** Schematic configuration of γ-GaS polarized imaging system.

**Supporting Information S17**

**Stress distribution measurement of a transparent lens**

**Figure S17**. Internal stress distribution measurement of a transparent lens measured by our γ-GaS detectors. *θ=*0°, 45°, 90°, and 135° is the angle between the polarization state of the incident light and the clockwise rotation of the material’s b-axis.

**Supporting Information S18**

**Digital image recognitions based on convolutional neural network**

**Figure S18.** (a) Sine relationship fitting between the photocurrent value and the polarization angle of the polarized light. (b, c) Relationship between the number of training times and the recognition accuracy (b)/loss (c). (d) Decryption images.

**References**

[1] E. D. Palik, *Academic Press* **1998**, 3.

[2] W. Shen, C. Hu, S. Huo, Y. Li, X. Hu, *Appl. Surf. Sci.* **2017**, 421, 535.

[3] Y. Wang, Z. Nie, F. Wang, *Light Sci. Appl.* **2020**, 9, 192.

[4] R. R. Nair, P. Blake, A. N. Grigorenko, K. S. Novoselov, T. J. Booth, T. Stauber, N. M. R. Peres, A. K. Geim, *Science* **2008**, 320, 5881.

[5] R. Bai, T. Xiong, J. Zhou, Y. Liu, W. Shen, C. Hu, F. Yan, K. Wang, D. Wei, J. Li, J. Yang, Z. Wei, *InfoMat* **2022**, 4, e12258.

[6] Q. Qin, W. Gao, H. Zhang, J. Chen, Y. Yan, K. Zhu, M. Long, G. Li, S. Yin, Y. Du, H. Zhang, Q. Wang, Z. Wang, Y. Li, S. Wang, L. Li, *J. Mater. Chem. A* **2023**, 11, 11517.

[7] J. Lai, X. Liu, J. Ma, Q. Wang, K. Zhang, X. Ren, Y. Liu, Q. Gu, X. Zhuo, W. Lu, Y. Wu, Y. Li, J. Feng, S. Zhou, J. Chen, D. Sun, *Adv. Mater.* **2018**, 30, 1707152.

[8] G. Li, H. Zhang, Y. Li, S. Yin, X. Kan, W. Wei, H. Du, B. Ge, C. An, M. Tian, F. Yan, S. Yang, T. Zhai, L. Li, *Nano Res.* **2022**, 15, 5469.

[9] Z. Liang, X. Zhou, L. Zhang, X. Yu, Y. Lv, X. Song, Y. Zhou, H. Wang, S. Wang, T. Wang, P. P. Shum, Q. He, Y. Liu, C. Zhu, L. Wang, X. Chen, *Nat. Commun.* **2023**, 14, 39995.

[10] X. Wang, K. Wu, M. Blei, Y. Wang, L. Pan, K. Zhao, C. Shan, M. Lei, Y. Cui, B. Chen, D. Wright, W. Hu, S. Tongay, Z. Wei, *Adv. Electron. Mater.* **2019**, 5, 1900419.

[11] K. Zhao, J. Yang, M. Zhong, Q. Gao, Y. Wang, X. Wang, W. Shen, C. Hu, K. Wang, G. Shen, M. Li, J. Wang, W. Hu, Z. Wei, *Adv. Funct. Mater.* **2020**, 30, 2006601.

[12] H. Zhang, Y. Li, X. Hu, J. Xu, L. Chen, G. Li, S. Yin, J. Chen, C. Tan, X. Kan, L. Li, *Appl. Phys. Lett.* **2021**, 119, 171102.

[13] X. Chen, W. Mu, Y. Xu, B. Fu, Z. Jia, F. Ren, S. Gu, R. Zhang, Y. Zheng, X. Tao, J. Ye, *ACS Appl. Mater. Interfaces* **2019**, 11, 7131.

[14] H. Yuan, X. Liu, F. Afshinmanesh, W. Li, G. Xu, J. Sun, B. Lian, A. G. Curto, G. Ye, Y. Hikita, Z. Shen, S. Zhang, X. Chen, M. Brongersma, H. Y. Hwang, Y. Cui, *Nat. Nanotechnol.* **2015**, 10, 707.

[15] A. Singh, X. Li, V. Protasenko, G. Galantai, M. Kuno, H. (G.) Xing, D. Jena, *Nano Lett.* **2007**, 7, 2999.

[16] M. Luo, F. Ren, N. Gagrani, K. Qiu, Q. Wang, L. Yu, J. Ye, F. Yan, R. Zhang, H. H. Tan, C. Jagadish, X. Ji, *Adv. Opt. Mater.* **2020**, 8, 2000514.

[17] L. Liu, H. Liang, Y. Huang, C. Cai, W. Liu, X. Yu, J. Zhang, *Opt. Express* **2024**, 32, 13128.

[18] X. Meng, Y. Du, W. Wu, N. B. Joseph, X. Deng, J. Wang, J. Ma, Z. Shi, B. Liu, Y. Ma, F. Yue, N. Zhong, P. Xiang, C. Zhang, C. Duan, A. Narayan, Z. Sun, J. Chu, X. Yuan, *Adv. Sci.* **2023**, 10, 2300413.

[19] W. Gan, Y. Liu, X. Liu, R. Xiao, K. Ni, M. Jiang, H. Han, X. Zhou, S. Li, C. Wu, Y. Li, H. Li, *ACS Appl. Mater. Interfaces* **2024**, 16, 24943.

[20] X. Fang, Y. Bando, M. Liao, U. K. Gautam, C. Zhi, B. Dierre, B. Liu, T. Zhai, T. Sekiguchi, Y. Koide, D. Golberg, *Adv. Mater.* **2009**, 21, 2034.

[21] X. Fang, S. Xiong, T. Zhai, Y. Bando, M. Liao, U. K. Gautam, Y. Koide, X. Zhang, Y. Qian, D. Golberg, *Adv. Mater.* **2009**, 21, 5016.

[22] Y. Tao, J. Chen, J. Wu, Y. Wu, X. Wu, *J. Alloys Compd.* **2015**, 646, 532.

[23] W. Xiong, J. Chen, X. Wu, J. Zhu, *J. Mater. Chem. C* **2015**, 3, 1929.

[24] P. Hu, L. Wang, M. Yoon, J. Zhang, W. Feng, X. Wang, Z. Wen, J. C. Idrobo, Y. Miyamoto, D. B. Geohegan, K. Xiao, *Nano Lett.* **2013**, 13, 1649.

[25] S. Yang, Y. Li, X. Wang, N. Huo, J. Xia, S. Li, J. Li, *Nanoscale* **2014**, 6, 2582.

[26] G. W. Mudd, S. A. Svatek, L. Hague, O. Makarovsky, Z. R. Kudrynsky, C. J. Mellor, P. H. Beton, L. Eaves, K. S. Novoselov, Z. D. Kovalyuk, E. E. Vdovin, A. J. Marsden, N. R. Wilson, A. Patane, *Adv. Mater.* **2015**, 27, 3760.

[27] Y. Endo, Y. Sekine, Y. Taniyasu, *Appl. Phys. Lett.* **2025**, 126, 043105.

[28] J. Liu, Y. Zhou, P. Shao, N. Ma, L. Jia, Y. Wang, H. Zhao, C. Lu, X. Xu, *Adv. Funct. Mater.* **2025**, 35, 2413123.

[29] Z. Li, D. Zhang, J. Li, J. Zhao, H. Qiao, X. Ren, Z. Huang, X. Qi, J. Zhong, S. C. Dhanabalan, H. Zhang, *Adv. Funct. Mater.* **2018**, 28, 1705237.

[30] Q. You, J. Zhu, C. Peng, Y. Zhang, B. Ruan, X. Dai, X. Wang, W. Wong, D. Xue, Y. Xiang, *Sci. China Tech. Sci.* **2022**, 65, 2297.

[31] M. I. Zappia, G. Bianca, S. Bellani, N. Curreli, Z. Sofer, M. Serri, L. Najafi, M. Piccinni, R. Oropesa-Nuñez, P. Marvan, V. Pellegrini, I. Kriegel, M. Prato, A. Cupolillo, F. Bonaccorso, *J. Phys. Chem. C* **2021**, 125, 11857.

[32] S. R. Tamalampudi, Y. Lu, R. Kumar, R. Sankar, C. Liao, K. Moorthy, C. Cheng, F. C. Chou, Y. Chen, *Nano Lett.* **2014**, 14, 2800.

[33] W. Feng, J. Wu, X. Li, W. Zheng, X. Zhou, K. Xiao, W. Cao, B. Yang, J. Idrobo, L. Basile, W. Tian, P. Tan, P. Hu, *J. Mater. Chem. C* **2015**, 3, 7022.

[34] Y. Cao, K. Cai, P. Hu, L. Zhao, T. Yan, W. Luo, X. Zhang, X. Wu, K. Wang, H. Zheng, *Sci. Rep.* **2015**, 5, 8130.

[35] F. Liu, H. Shimotani, H. Shang, T. Kanagasekaran, V. Zolyomi, N. Drummond, V. I. Fal'ko, K. Tanigaki, *ACS Nano* **2014**, 8, 752.

[36] W. Feng, F. Qin, M. Yu, F. Gao, M. Dai, Y. Hu, L. Wang, J. Hou, B. Li, P. Hu, *ACS Appl. Mater. Interfaces* **2019**, 11, 18511.

[37] Y. Lu, J. Chen, T. Chen, Y. Shu, R. Chang, Y. Sheng, V. Shautsova, N. Mkhize, P. Holdway, H. Bhaskaran, J. H. Warner, *Adv. Mater.* **2020**, 32, 1906958.

[38] S. Lei, L. Ge, Z. Liu, S. Najmaei, G. Shi, G. You, J. Lou, R. Vajtai, P. M. Ajayan, *Nano Lett.* **2013**, 13, 2777.

[39] M. Song, N. An, Y. Zou, Y. Zhang, W. Huang, H. Hou, X. Chen, *Front. Phys.* **2023**, 18, 52302.

[40] Z. Guo, R. Cao, H. Wang, X. Zhang, F. Meng, X. Chen, S. Gao, D. K. Sang, T. H. Nguyen, A. T. Duong, J. Zhao, Y. Zeng, S. Cho, B. Zhao, P. Tan, H. Zhang, D. Fan, *Natl. Sci. Rev.* **2021**, 8, nwab098.

[41] X. Li, M. Lin, A. A. Puretzky, J. C. Idrobo, C. Ma, M. Chi, M. Yoon, C. M. Rouleau, I. I. Kravchenko, D. B. Geohegan, K. Xiao, *Sci. Rep.* **2014**, 4, 5497.

[42] P. Hu, Z. Wen, L. Wang, P. Tan, K. Xiao, *ACS Nano* **2012**, 6, 5988.

[43] W. Zhong, Y. Liu, X. Yang, C. Wang, W. Xin, Y. Li, W. Liu, H. Xu, *Mater. Des.* **2021**, 212, 110233.

[44] F. Xia, H. Wang, Y. Jia, *Nat. Commun.* **2014**, 5, 4458.

[45] E. Liu, Y. Fu, Y. Wang, Y. Feng, H. Liu, X. Wan, W. Zhou, B. Wang, L. Shao, C. Ho, Y. Huang, Z. Cao, L. Wang, A. Li, J. Zeng, F. Song, X. Wang, Y. Shi, H. Yuan, H. Y. Hwang, Y. Cui, F. Miao, D. Xing, *Nat. Commun.* **2015**, 6, 6991.

[46] H. Liu, G. Luo, H. Cheng, Z. Yang, Z. Xie, K. H. L. Zhang, Y. Yang, *J. Phys. Chem. Lett.* **2022**, 13, 4988.

[47] N. Huo, G. Konstantatos, *Adv. Mater.* **2018**, 30, 1801164.

[48] H. Fang, W. Hu, *Adv. Sci.* **2017**, 4, 1700323.

[49] Z. Guo, J. S. Manser, Y. Wan, P. V. Kamat, L. Huang, *Nat. Commun.* **2015**, 6, 7471.
